# Measured but Not Induced Perspective-taking Predicts Success in Coalition Formation

**DOI:** 10.1177/01461672251349706

**Published:** 2025-07-14

**Authors:** Anabela Cantiani, Ilja van Beest, Thorsten M. Erle

**Affiliations:** 1 Tilburg University, Netherlands

**Keywords:** perspective-taking, mentalizing, social cognition, trait-state, coalition formation, horizontal collaboration

## Abstract

Reducing CO_2_ emissions in the transport sector is a societal challenge that requires collaboration, such as sharing cargo space to reduce truck usage. Yet, such collaboration remains rare due to self-interest. A recent study using a game simulating transport collaboration showed that measured perspective-taking (focusing on others) predicted greater inclusion in collaborations and higher earnings. In two experiments (*N* = 1,446), we experimentally induced perspective-taking for one party before the negotiation to test for comparable effects. While both experiments replicated the effects of measured perspective-taking in behaviors conducive to CO_2_ reduction (proposing larger coalitions and making more other-serving offers), neither replicated the economic benefits. This suggests that while both measured and induced perspective-taking influence coalition behavior similarly, only measured perspective-taking predicts favorable economic outcomes. Further analyses showed that experimentally inducing perspective-taking only leads to beneficial outcomes if participants actively engage in it. Potential explanations for this discrepancy are discussed.

Major societal challenges like climate change can only be solved via collaborative efforts. One notable opportunity for this is the transport industry, which accounts for nearly 24% of global CO_2_ emissions from fuel combustion (International Energy Agency, 2020). Thus, to achieve the climate goals under the Paris Agreement, it is crucial for the transport industry to adopt more sustainable practices.

The International Transport Forum (2021) identifies horizontal collaboration as an effective strategy to reduce emissions, encouraging companies to share cargo space and thereby reduce the number of trucks on the road. For example, despite being competitors, Nestlé and PepsiCo collaborated horizontally by aligning their distribution networks and consolidating their shipments (Nestlé, 2014). Nestlé’s products were so heavy that a truck at weight capacity could only load about 50% of its space. PepsiCo’s products, on the other hand, could fill the cargo space without reaching the weight capacity. By sharing trucks to transport their products, this collaboration not only led to economic benefits but also fewer vehicles on the road, and thus lower carbon emissions. Despite the environmental and economic benefits and existing global agreements aimed at reducing carbon footprints, such collaborative initiatives remain rare in transport (Cruijssen et al., 2023). To foster these initiatives, it is crucial to understand the factors that promote horizontal collaboration and how people engage in this process.

From a social psychology perspective, horizontal collaboration can be conceptualized as a coalition formation process, where two or more parties pool their resources to obtain a mutually desired outcome (e.g., reduced carbon emissions, financial savings, increased power; Komorita & Kravitz, 1983). However, major coalition formation theories, which solely focus on economic aspects of the process, fail to account for psychological and other factors that promote coalition formation independently of economic motives.

Some empirical studies have begun to address this gap by examining how perspective-taking, the ability to consider other people’s mental states, affects cooperative coalition behavior (Van Beest et al., 2005, 2011). Of particular relevance is a recent study using a coalition formation paradigm that simulates horizontal transport collaboration, which found that individuals who reported higher levels of perspective-taking during the coalition negotiations were more likely to be included in winning coalitions, received higher payoffs, and crucially, proposed more inclusive (i.e., larger) collaborations that could lead to greater CO_2_ reductions (Cantiani, Van Beest, Cruijssen, et al., 2024).

One limitation of this study was that it only measured perspective-taking. While it is valuable to learn that a trait-like construct such as perspective-taking predicts sustainability and profits, it is first unclear whether perspective-taking causes these beneficial outcomes. Second, relying on stable individual dispositions limits the power of potential interventions. However, perspective-taking is both seen as an individual disposition and a state that can be situationally induced. In the present study, we therefore experimentally induce perspective-taking in a coalition formation context. By doing so, we aim to establish a causal link between perspective-taking and collaborative behaviors and to explore the potential of state perspective-taking inductions as a tool to increase horizontal collaboration and reduce CO_2_ emissions.

## Perspective-taking

Perspective-taking is the ability to process and understand other people’s thoughts, feelings, and perceptions. Perspective-taking is fundamental to social understanding, coordination, and effective communication, enabling people to navigate complex social environments (Arriaga et al., 1998; Mead, 1934; Galinsky et al., 2005, 2014; Novembre et al., 2019).

Research has shown that perspective-taking offers a range of benefits in dyadic negotiations and social decision-making settings that involve both cooperation and competition. In integrative or “win-win” negotiations, perspective-taking allows negotiators to better understand the interests, motivations, and constraints of their counterparts, enabling agreements that maximize both individual and collective gains (Galinsky et al., 2008; Gilin et al., 2013; Neale & Bazerman, 1983). This ability also helps negotiators to avoid full and partial impasses (Trötschel et al., 2011) by increasing insight into the other party’s position, by improving information exchange between parties (Galinsky et al., 2014; Homburg et al., 2009), and by reducing cognitive biases (e.g., anchoring effects or self-serving biases), which often hinder productive negotiations (Galinsky & Mussweiler, 2001; Drolet et al., 1998).

Importantly, perspective-taking can affect negotiations both when conceptualized as a *measured trait* and an *induced state*. For example, Galinsky et al. (2008) demonstrated the effects of perspective-taking both measured and induced through situational instructions. In a buyer–seller negotiation over a gas station, higher trait perspective-taking significantly predicted more successful outcomes (i.e., whether a deal meeting both sides’ needs was reached). Similarly, state perspective-taking induced via instructions increased the likelihood of closing a deal (76%) compared to a control condition (39%). This dual nature of perspective-taking, as both a measured trait and an induced state, makes it an attractive target for interventions aimed at promoting cooperative behaviors, such as those needed to reduce CO_2_ emissions in transport.

## The Role of Perspective-taking in Coalition Formation

Although perspective-taking is fundamental to social interaction, economists have largely neglected its role in decision-making (Cruijssen et al., 2023). This omission is particularly surprising given that core concepts in Game Theory, such as Nash equilibria, backward induction, and the iterated elimination of dominated strategies, rely on the implicit assumption that individuals can accurately grasp others’ perspectives, preferences, and beliefs (Singer & Fehr, 2005). However, empirical research demonstrates that perspective-taking it is highly variable and influenced by context (Chopik et al., 2017; Will & Güroğlu, 2016).

This gap extends to major theories of coalition formation, which are built on game-theoretic foundations (Kahan & Rapoport, 1984). These theories aim to predict outcomes, such as which coalitions will form and how payoffs will be divided, based solely on game parameters (Komorita & Kravitz, 1983; Murnighan, 1991). As a result, they disregard the (psychological) mechanisms that shape players’ behavior (Van Beest & Van Dijk, 2007). Even social psychological models,^1^ often called equity theories, which attempt to integrate n-person game theory with insights from social psychology, still operate within the rational choice paradigm.^2^ Although they incorporate factors like social norms and power dynamics (Murnighan, 1978; Kahan & Rapoport, 1984), these models continue to assume that players have perfect perspective-taking abilities and can accurately predict others’ strategies based on shared game information (Cantiani, Van Beest, Cruijssen, et al., 2024; Cantiani, Van Beest, & Erle, 2024).

To illustrate how coalition theories implicitly rely on perspective-taking, consider the Strength-is-Weakness (SiW) effect, a paradox in which having more resources becomes a disadvantage in coalition formation (Murnighan, 1991; Vinacke & Arkoff, 1957; Wissink et al., 2021, 2022a). This effect typically emerges in experimental tasks known as weighted majority games. In these games, each player is assigned a different number of resources (e.g., Player A has 4, Player B has 3, and Player C has 2) and they must form coalitions that together reach a minimum threshold (e.g., 5 points) to win a collective reward. According to minimum resource theory, players’ preferences are determined by these resource weights, and an equity norm is presumed to guide payoff allocation (i.e., players expect payoffs to be proportional to their contributed resources). Players with fewer resources anticipate that stronger players will demand a larger share of the payoff due to their higher contributions. To avoid these demands, weaker players form a winning coalition that excludes the strongest player.

For the SiW effect to occur, players must engage in perspective-taking, that is, they must infer how others interpret game information and which allocation norms they will apply. Typically, players expect others to focus on resource weights and adopt an equity norm, demanding payoffs proportional to their contributions. This mental state inference is central to coalition decisions and, implicitly, to the predictions of coalition theories. However, these theories assume that all players can accurately anticipate others’ preferences and adjust accordingly. If this were true, strong players would adjust their demands to avoid exclusion. Yet, the continued presence of the SiW effect suggests otherwise. Not all strong players are excluded, and not all fail to adapt, indicating variation in perspective-taking. This variation may explain why some players adjust their strategies and derive more favorable outcomes.

Initial support for the role of perspective-taking in coalition formation has been provided in a recent study (Cantiani, Van Beest, Cruijssen, et al., 2024). The researchers developed *The Transport Game*, a 5(4-3-2) weighted majority game that simulates horizontal collaboration in the transport sector. In the game, three companies with differing transport volumes (4, 3, and 2 tons) had to form coalitions to meet a minimum cargo threshold of 5 tons, enabling them to use a shared truck. Participants negotiated to form coalitions and decide how to split a monetary reward. Perspective-taking was assessed through two self-report measures. The first was the Interpersonal Reactivity Index (IRI) perspective-taking subscale (Davis, 1980), which captures broad, cross-situational tendencies (general perspective-taking). The second was a version adapted to the negotiation setting (contextualized perspective-taking), designed to assess the activation of perspective-taking within the task. This reflects what the literature describes as trait expression in context (Fleeson, 2001; Fleeson & Jayawickreme, 2015).

This distinction was supported empirically: Only contextualized perspective-taking predicted favorable negotiation outcomes. Hereafter, we refer to the contextualized version simply as measured perspective-taking, unless otherwise specified. Individuals higher on this measure were more frequently included in winning coalitions and secured larger shares of the bonus. Perspective-takers employed two key strategies. First, they more often proposed grand coalitions involving all three companies. This approach promotes more inclusive partnerships that can utilize resources more efficiently. Second, perspective-takers made more other-serving proposals, allocating more money to potential partners rather than maximizing their own gains. These negotiation strategies are particularly conducive to reducing CO_2_ emissions in transport, as they promote larger and more sustainable partnerships. Altogether, these findings emphasize the role of measured contextualized perspective-taking in coalition formation, challenging key assumptions of coalition theories. First, perspective-taking varies significantly across individuals, and this variation predicts negotiation success. Second, successful strategies often diverge from rational models that typically assume two-person coalitions and self-maximizing offers.^3^

Despite the promising findings of this study, there are two important reasons that warrant further research. Its correlational design limits causal inference, as it cannot rule out confounding variables that may drive the observed relationships. Moreover, while this study focused on perspective-taking as an individual disposition expressed in a given context, perspective-taking can also be induced as a temporary state. In dyadic negotiation research, both measured and induced perspective-taking have been shown to enhance outcomes, but their induced effects remain unexplored in coalition settings involving three or more parties. To address these gaps, the present research aims to establish causal relationships and explore whether induced perspective-taking can also predict success in coalition formation in a transport setting.

## The Present Research

To test whether perspective-taking can lead to coalition formation success, as well as an induced state, the present study aimed to experimentally manipulate perspective-taking. Otherwise, we closely replicated the methodology of previous work on the role of perspective-taking during coalition formation. Thus, participants again completed the Transport Game (Cantiani, Van Beest, Cruijssen, et al., 2024). Participants were organized into triads and randomly assigned to one of two conditions: perspective-taking or egocentrism. To manipulate perspective-taking, we used written instructions framed as a “performance tip.” Player As in the perspective-taking condition received instructions to consider other players’ perspectives during the negotiation, whereas player As in the other (egocentric) condition were instructed to focus on their own perspective throughout the negotiation. The remaining triad members (player Bs and Cs) received neutral instructions matched in length and complexity to the experimental manipulations, but neither emphasizing perspective-taking nor egocentrism.

We targeted only player As for this manipulation, as they hold the most resources, but face a structural disadvantage in coalition formation: the SiW effect. Due to this, player Bs and Cs are already frequently included in winning coalitions, and there is limited room for improving their inclusion. In contrast, player As’ lower baseline inclusion rates offer a stronger test for the benefits of induced perspective-taking.

We hypothesized that player As in the perspective-taking condition would (H1) be included in more winning coalitions and (H2) obtain higher payoffs than those in the egocentric condition. We also explored whether induced perspective-taking promotes the same specific negotiation behaviors as measured perspective-taking, that is, proposing larger coalitions and making more other-serving proposals.

By experimentally inducing perspective-taking, this research aims to establish its causal role in coalition formation and to test whether temporary state-level interventions can replicate the benefits of contextually activated traits. In doing so, the study contributes both theoretically and practically to the fields of coalition formation and perspective-taking research. These insights also have practical implications, as they relate to real-world issues like improving collaboration on global challenges such as reducing CO_2_ emissions in transport.

## Open Practices, Power Analysis, and Ethics

All de-identified data, analyses scripts, codebooks, and materials are available at https://osf.io/4xkjy/. All design and analytical decisions reported in this manuscript were preregistered (Experiment 1: https://osf.io/s8kzm; Experiment 2: https://osf.io/wqt9m). We report all manipulations, measures, and exclusions, and deviations from our preregistrations (only once in Experiment 2). Excluding participants who did not correctly answer any comprehension, attention checks, or manipulation checks in either experiment did not affect any of the results.

The sample size for both experiments was determined through a power simulation using the rbinom package in R. A pilot study with *N* = 141 players (47 triads) showed that player A was in 55.17% of winning coalitions under perspective-taking but only 34.48% in the egocentric conditions. To detect a significant difference of this size with 90% power in a logistic regression, at least 120 participants per condition were needed, totaling 240 player As. The total required sample was thus 720 participants (including also 240 player Bs and Cs each).

All experiments were approved by the Ethics Review Board of the School of Social and Behavioral Sciences of Tilburg University.

## Experiment 1

### Method

#### Participants

We recruited 726 participants through Prolific Academic (*M*_age_ = 35.3 years, *SD* = 11.6), resulting in 242 triads. The sample consisted of 51.4% (*n* = 371) women, 47.9% (*n* = 346) men, 0.4% (*n* = 3) nonbinary individuals, and 0.3% (*n* = 2) who preferred not to say. Triads were randomly assigned to either the *perspective-taking* condition (122 triads) or the *egocentric* condition (120 triads). Eligible participants were required to be over 18 years old, to be fluent in English, and to have a 95% approval rate on Prolific. Recruitment occurred in batches of 30 participants to maximize the possibility of real-time matching. Participants received £2.50 for completing the task and another £0.28 per 1,000 Euros they earned in the game, leading to a payout of between £2.50 and £5.00.

#### Materials

##### The Transport Game

To be able to have three participants interactively negotiate about forming a coalition in real time, participants completed the “Transport Game” (Cantiani, Van Beest, Cruijssen, et al., 2024). This game is a 5(4-3-2) simple weighted majority game (Murnighan, 1978, 1991), developed in oTree (Chen et al., 2016) and set in a transport context. In this scenario, they assumed the roles of transport companies (A, B, and C) tasked with moving goods from Amsterdam to Paris. Players were informed that the companies varied in size and used individual trucks to transport their own freight. Company A usually carries 4 tons of goods, Company B 3 tons, and finally Company C 2 tons.

Within the game, there is a large truck capable of carrying up to 9 tons of cargo, which the companies could use to bundle their loads. However, this truck was only permitted on the road if it carried at least 5 tons. Thus, the formation of a coalition between at least two companies is needed to use the large truck. An additional incentive was provided: Another company needed the large truck to transport goods back to Amsterdam from Paris, offering a premium of 9,000 Euros to the companies that brought it to Paris. Any coalition of companies that could provide at least 5 tons of freight could utilize the large truck to carry their cargo to Paris and claim the bonus.

**Figure 1.**
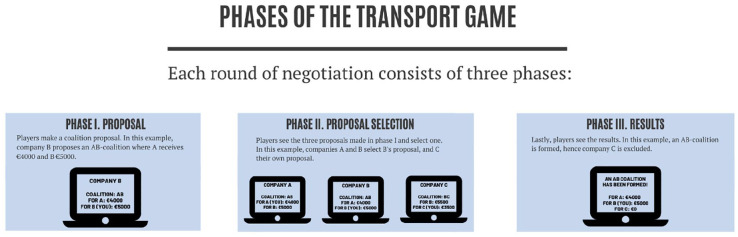
Negotiation protocol of the transport game.

The Transport Game consisted of three phases, detailed in Figure 1. These three phases repeated until either a coalition was formed, or for 20 rounds, after which the negotiation ended without the formation of a coalition. The three phases were:

Phase I: Players indicated their preferred coalition (AB, BC, AC, or ABC) and proposed how to distribute the 9,000 Euro bonus among coalition members.
Phase II: All offers from Phase I were presented to all players, and they could select their preferred proposal (of those involving themselves).
Phase III: Players saw the selections made by others. If all members of a proposed coalition chose the same offer, that coalition was formed, and the negotiation ended. Otherwise, no coalition was formed, and a new round of negotiations began.

##### Manipulated Variables

**Figure 2.**
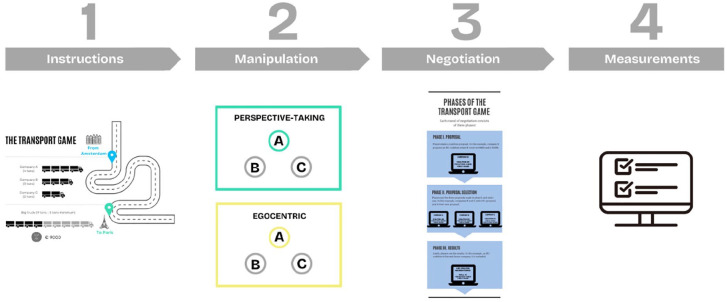
Overview of experimental phases.

The manipulation was implemented after players had learned the game scenario and successfully answered comprehension questions, but immediately before the start of negotiations (see Figure 2). Players received specific instructions framed as a “performance tip” for the upcoming coalition negotiation, adapted from previous research by Gilin et al. (2013) and Trötschel et al. (2011). Lastly, to reinforce the manipulation, following Gilin et al. (2013), we asked all players to write a short essay about their “performance tip.” We only introduced minor adjustments to ensure that the word count and complexity were as similar as possible across player As and conditions. The full materials, including detailed instructions and manipulations, can be found in Supplemental Material A.

##### Measured Variables

After the completion of the game, we assessed several variables to evaluate the effectiveness of our manipulation and for the main analyses. These variables were presented to participants in the order described below.

As a first manipulation check, participants selected their assigned “performance tip” in a multiple-choice format. As a second manipulation check, participants completed the IRI perspective-taking and empathic concern subscales (Davis, 1980). The perspective-taking subscale assesses a general disposition to spontaneously adopt others’ psychological viewpoints across diverse situations and social targets, while the empathic concern subscale measures feelings of sympathy and concern for others facing unfortunate circumstances. An attention check item was embedded within the empathic concern subscale, instructing participants to select response option “4.” These dispositional scales served as control measures, which we expected to remain stable and unaffected by the experimental manipulation, consistent with Whole Trait Theory (Fleeson & Jayawickreme, 2021), which holds that global traits represent stable averages over time. In contrast, participants also completed a contextualized perspective-taking scale (Cantiani, Van Beest, Cruijssen, et al., 2024), designed to capture momentary, situational activations of perspective-taking specifically within the coalition formation task. This scale reflects the state-like expression of trait perspective-taking as it fluctuates with task demands. Accordingly, we predicted player As in the perspective-taking/egocentric conditions to score higher/lower than player Bs and Cs in their respective conditions. In addition, we predicted player As in the perspective-taking condition to score higher than player As in the egocentric condition.

Additional measures included perceived satisfaction (Galinsky et al., 2008), fundamental need satisfaction (Williams, 2009), and perceived similarity to other players (Aron et al., 1992). Demographic data were also collected. All measures and instructions can be found in Supplemental Material B.

#### Procedure

After providing informed consent, participants were informed that they would negotiate to attain a sum of money with two other players in real time. A detailed explanation of the rules of the Transport Game followed. They were told that their decisions would translate into monetary rewards. To ensure timely progression, some pages had 5 min timers. Participants were warned that failing to complete a page within the time limit would terminate the study for the entire triad, resulting in only a base payment for all participants. They were then grouped into triads based on arrival order and randomly assigned to a company (A, B, or C). Once participants had read the instructions, but before starting the negotiation process, they were quizzed on their comprehension of the game (see Supplemental Material A). Upon successfully passing the comprehension checks, participants received a “performance tip,” that is, the manipulation of this study. After completing the negotiation and all subsequent measures, participants were debriefed about the study’s purpose.

## Results

Data were analyzed using R, version 4.4.2 (R Core Team, 2024).

### Manipulation and Comprehension Checks

We assessed participants’ instruction comprehension and the effectiveness of the experimental manipulation in multiple ways. Most participants demonstrated strong comprehension, and manipulation checks confirmed that player As in the perspective-taking condition engaged in perspective-taking more than player As in the egocentric condition. Full descriptive statistics and analyses of these checks are reported in Supplemental Material B.

### Coalition Behavior

In the following analyses, player A’s condition (perspective-taking vs. egocentric) was used as the predictor for the dependent variables derived from the coalition game, which are detailed in the subsequent sections.

#### Partner-related Initial Behavior of Player A: Coalition Size

To test whether perspective-takers approached more players in their first proposals (as in Cantiani, Van Beest, Cruijssen, et al., 2024), we analyzed coalition size as a dependent variable (coded as 1 for *grand coalition* [i.e., ABC] proposals, and 0 for *small coalition* [i.e., AB, or AC] proposals). As predicted, player As in the perspective-taking condition proposed more grand coalitions (48.4%) than those in the egocentric condition (28.3%), *OR* = 2.37, 95% CI [1.40, 4.06], *z* = 3.17, *p* = .002. The deviance test comparing the null model to the model including the condition predictor indicated that its addition improved the model fit, *X*^2^(1, 241) = 10.36, *p* = .001, *R*^2^ = .03.

#### Allocation-related Initial Behavior of Player A: Self-allocated Money

To test whether perspective-takers made less self-serving opening proposals (as in Cantiani, Van Beest, Cruijssen, et al., 2024), we used the amount of money player As allocated to themselves when making a first offer as the dependent variable. As predicted, player As in the perspective-taking condition allocated less money to themselves (*M* = 4,778 Euros, *SD* = 1,245) than those in the egocentric condition (*M* = 5,176 Euros, *SD* = 1,198), β = −397.62, 95% CI [−707.09, −88.15], *t*(240) = −2.53, *p* = .012. The regression model was statistically significant, *F*(1, 240) = 6.41, *p* = .012, *R*^2^ = .03.

Table 1 summarizes these results and shows that inducing perspective-taking leads to the same coalition behaviors as measured perspective-taking (Cantiani, Van Beest, Cruijssen, et al., 2024): Either form of perspective-taking leads individuals to approach more partners during negotiations and to make less self-serving offers.

**Table 1.** Initial Behavior: Frequency of Opening Proposals and Associated Offers.

Position	Perspective-taking condition								
	Coalition	*N*	%	*M*_A_	(*SD*)	*M*_B_	(*SD*)	*M*_C_	(*SD*)
A (4 tons)	AB (7 tons)	12	9.8	5,254.2	(388.1)	3,745.8	(388.1)	—	—
	AC (6 tons)	51	41.8	5,796.1	(734.3)	—	—	3,203.9	(734.3)
	ABC (9 tons)	59	48.4	3,802.1	(901.5)	2,959.0	(411.6)	2,238.9	(630.5)
B (3 tons)	AB (7 tons)	9	7.4	5,061.1	(390.3)	3,938.9	(390.3)	—	—
	BC (5 tons)	83	68.0	—	—	5,377.7	(717.9)	3,622.3	(717.9)
	ABC (9 tons)	30	24.6	3,550	(497.4)	3,050	(201.3)	2,400	(563.2)
C (2 tons)	AC (7 tons)	16	13.2	4,750	(1,602)	—	—	4,250	(1,602)
	BC (6 tons)	68	55.7	—	—	5,072.8	(382.5)	3,927.2	(382.5)
	ABC (9 tons)	38	31.1	3,723.7	(720.8)	2,946.1	(226.7)	2,330.2	(765.9)
	Egocentric condition								
	Coalition	*N*	%	*M*_A_	(*SD*)	*M*_B_	(*SD*)	*M*_C_	(*SD*)
A (4 tons)	AB (7 tons)	15	12.5	5,508.7	(773.9)	3,491.3	(773.9)	—	—
	AC (6 tons)	71	59.2	5,854.9	(726.3)	—	—	3,145.1	(726.3)
	ABC (9 tons)	34	28.3	3,611.8	(489.7)	3,002.9	(17.15)	2,385.3	(491.2)
B (3 tons)	AB (7 tons)	17	14.2	4,779.8	(445.9)	4,220.1	(445.9)	—	—
	BC (5 tons)	79	65.8	—	—	5,234.1	(511.6)	3,765.8	(511.6)
	ABC (9 tons)	24	20.0	3,541.7	(508.9)	3,000.0	(0)	2,458.3	(508.9)
C (2 tons)	AC (7 tons)	8	6.7	5,412.5	(689.6)	—	—	3,587.5	(689.6)
	BC (6 tons)	66	55.0	—	—	4921.2	(683.5)	4,078.8	(683.5)
	ABC (9 tons)	46	38.3	3,578.3	(505.0)	2,958.7	(168.1)	2,463.0	(535.1)

### Coalition Outcomes

#### Inclusion in Winning Coalitions

**Figure 3.**
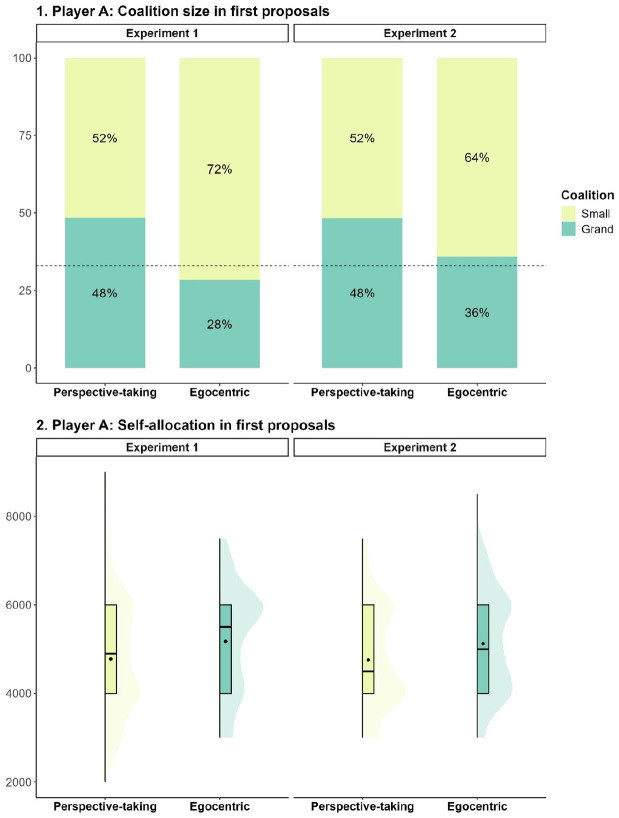
Initial behavior of player A in experiments 1 and 2. *Note.* Error bars indicate the 95% confidence intervals. Lines within the box plots indicate the median. Dots within the box plots indicate the mean.

Inclusion in winning coalitions was dummy coded as 1 when player As were included in formed coalitions, or 0 when player As were excluded. Contrary to our predictions, as illustrated in Figure 3, the inclusion rates of player As did not differ between the perspective-taking (41.0%) and the egocentric (49.2%) conditions, *OR* = 0.72, 95% CI [0.43, 1.19], *z* = −1.28, *p* = .201. The deviance test comparing the null model with the model including the condition predictor was not significant, *X*^2^(1, 241) = 1.64, *p* = .201, *R*^2^ = .01.

#### Final Payoffs

To test this prediction, we used the final amount of money players received at the end of the negotiation as the dependent variable. Contrary to our hypothesis, as shown in Figure 3, there were no significant differences in final payoffs between player As in the perspective-taking (*M* = 1,760 Euros, *SD* = 2,196) and egocentric conditions (*M* = 2,192 Euros, *SD* = 2,338), β = −432.30, 95% CI [−1,006.50, 141.99], *t*(240) = −1.48, *p* = .139. The multiple regression model did not explain a significant proportion of variance, *F*(1, 240) = 2.20, *p* = .139, *R*^2^ = .01.

Table 2 summarizes these results and shows that at the outcome level, inducing perspective-taking did not lead to the same advantages as measured perspective-taking in previous work (Cantiani, Van Beest, Cruijssen, et al., 2024): While measured perspective-taking predicted more inclusion in winning coalitions and higher payoffs, induced perspective-taking did not affect either of these variables.

**Table 2.** Coalition Outcomes: Frequency of Winning Coalitions and Associated Payoff.

Perspective-taking condition								
Coalition	*N*	%	*M*_A_	(*SD*)	*M*_B_	(*SD*)	*M*_C_	(*SD*)
AB (7 tons)	7	5.7	4,778.6	(264.3)	4,221.4	(264.3)	—	—
AC (6 tons)	23	18.9	4,882.6	(672.6)	—	—	4,117.4	(672.6)
BC (5 tons)	72	59.0	—	—	5,095.8	(554.5)	3,904.2	(554.5)
ABC (9 tons)	20	16.4	3,450.1	(510.4)	2,999.9	(0.4)	2,550.1	(510.5)
Egocentric condition								
Coalition	*N*	%	*M*_A_	(*SD*)	*M*_B_	(*SD*)	*M*_C_	(*SD*)
AB (7 tons)	9	7.5	4,816.6	(308.2)	4,183.3	(308.2)	—	—
AC (6 tons)	27	22.5	5,138.9	(721.7)	—	—	3,861.1	(721.7)
BC (5 tons)	61	50.8	—	—	5,071.3	(422.9)	3,928.7	(422.9)
ABC (9 tons)	23	19.2	3,521.7	(510.8)	3,000.0	(0.0)	2,478.3	(510.8)

### Exploratory Analyses

Since player As’ understanding of the situation and the approach to it was so similar between previous studies (Cantiani, Van Beest, Cruijssen, et al., 2024) and the present study, the following analyses aimed at finding out whether these differences in final outcomes were caused by variations in player Bs’ and Cs’ behavior between the two studies. To explore this, we compared the initial behaviors of player Bs and Cs in the current experiment to those observed before (Cantiani, Van Beest, Cruijssen, et al., 2024).

#### Partner-related Initial Behavior of Players B and C: Coalition Size

A Pearson’s Chi-squared test was conducted on the proportions of proposed coalitions (BC coalition vs. other coalitions, i.e., AC, AB, and ABC) between studies (Correlational vs. Experimental). We found that presently, player Bs and Cs proposed more BC coalitions (61.2%) than player Bs and Cs in previous studies (50.8%), χ²(1) = 8.86, *p* = .003.

#### Allocation-related Initial Behavior of Players B and C: Self-allocated Money

**Figure 4.**
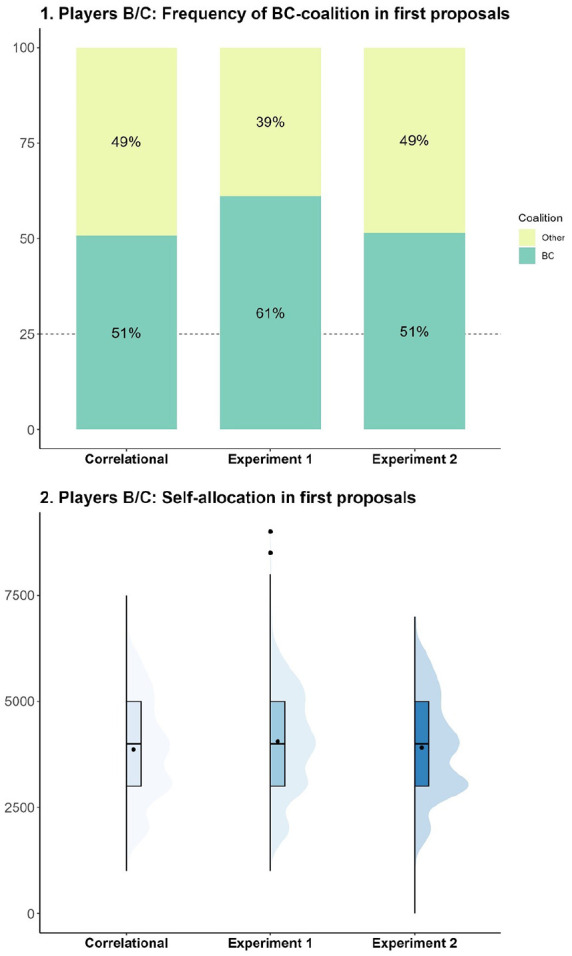
Comparison of Player B’s and C’s initial behavior between studies. *Note.* Error bars indicate the 95% confidence intervals. Lines within the box plots indicate the median. Dots within the box plots indicate the mean.

A linear regression model with study (Correlational vs. Experimental) as predictor of self-allocated money in first proposals of player Bs and Cs showed that in the current experiment they allocated significantly more money to themselves (*M* = 4,061 Euros, *SD* = 1,230; correlational study: *M* = 3,867 Euros, *SD* = 1,193), β = 193.60, 95% CI [30.04, 357.16], *t*(860) = 2.32, *p* = .020. The regression model was statistically significant, *F*(1, 860) = 5.40, *p* = .020, *R*^2^ = .01. Figure 4 summarizes these exploratory findings.

## Discussion

Participants in the perspective-taking condition accurately recalled their instructions, reported higher levels of perspective-taking, and displayed behaviors consistent with previous findings for measured perspective-taking (Cantiani, Van Beest, Cruijssen, et al., 2024). Specifically, they were more likely to approach more players with initial proposals and make more other-serving offers. However, despite these highly similar initial perceptions and behaviors, Experiment 1 did not find that induced perspective-taking increases inclusion rates or payoffs in coalition negotiations. We conducted another experiment to rule out alternative explanations for this difference.

The first explanation concerns the performance tips given to player Bs and Cs. The neutral instructions, intended to have no impact, may have inadvertently influenced their behavior. Unlike in the correlational study, where no tips were provided, player Bs and Cs in our experiment approached player A less frequently and allocated more money to themselves. It is possible that instructions such as “focus on your own approach to maximize outcomes” and “follow it consistently” encouraged more strategic and self-serving behavior, which could have undermined the intended effects of perspective-taking.

Second, and turning to player A, prior research has shown that in competitive environments, perspective-taking can sometimes backfire, resulting in detrimental rather than beneficial outcomes. Specifically, this has been attributed to a phenomenon known as reactive egoism, where perspective-takers make less selfish judgments but act more selfishly in practice (Epley et al., 2006). If player As exhibited reactive egoism in Experiment 1, we would observe that: (a) It occurs in a (perceived) competitive environment, but not in a cooperative one, (b) at the judgment level, perspective-takers make less egoistic (selfish) judgments than players in the egocentric conditions, but (c) at the behavioral level, perspective-takers behave more selfishly than players in the egocentric condition.

A third possible factor could be a difference between measured and induced perspective-taking. While measured perspective-taking captures individual dispositions to consider other’s mental states to anticipate what others might do during the game, induced perspective-taking aims to elicit this state through instructions, regardless of trait differences. According to Whole Trait Theory, people differ in their baseline tendency to produce such states. Participants with low measured perspective-taking, for example, may have found the instruction difficult or unfamiliar. This discrepancy could explain why some individuals did not benefit as expected from the perspective-taking manipulation.

## Experiment 2

To test these alternative explanations, Experiment 2 closely replicated Experiment 1, with two key differences. First, we removed the performance tips for player Bs and Cs, hoping to restore their initial behavior back to normal. Second, we introduced additional measures to assess the signs of reactive egoism and individual differences in induced perspective-taking performance. With these changes, we aimed to confirm our predictions for Experiment 1 not only on the level of comprehension and initial behavior, but also on the coalition outcome level.

## Method

### Participants

We recruited 720 participants through Prolific Academic, resulting in 240 triads (*n* = 120 triads in each condition). The sample consisted of 49.0% women (*n* = 350), 49.6% men (*n* = 355), 0.7% nonbinary individuals (*n* = 5), and 0.7% who preferred not to disclose their gender (*n* = 5). Participants’ ages ranged from 18 to 78 years (*M* = 39.2 years, *SD* = 12.3). The participant requirements and recruitment procedures were identical to Experiment 1.

### Procedure

Experiment 2 followed the same design as Experiment 1, with one key difference: Only player A received a performance tip. Thus, if our “neutral” performance tip indeed affected B’s and C’s behavior in Experiment 1, this would no longer be possible. Consequently, the first manipulation check was revised to ask only player A to recall the performance tip, with two response options corresponding to the perspective-taking or egocentric conditions. Although our preregistration stated asking all participants about the tip, we realized it was irrelevant for player Bs and Cs, who did not receive one.

To assess reactive egoism, we included measures of perceived competitiveness (Epley et al., 2006), fairness judgments (Epley et al., 2006) and motivations to maximize own outcomes, minimize harm to the other bargainers, and to make sure that every bargainer got what they deserved (Wissink et al., 2023). To assess perspective-taking ability, we added measures of objective perspective-taking performance. Specifically, we asked participants to estimate how many AB, AC, BC, ABC coalitions they thought would be formed by the sample during the transport game. For detailed descriptions of these measures, see Supplemental Material A.

## Results

Experiment 2 followed the same analytical strategies used in Experiment 1.

### Manipulation and Comprehension Checks

Comprehension and manipulation check results mirrored those of Experiment 1 and supported the effectiveness of our manipulation. As expected, only contextualized perspective-taking varied by condition and player role. Full results are reported in Supplemental Material B.

#### Initial Behavior of Players B and C

To verify whether removing the neutral instructions restored player Bs’ and Cs’ behavior back to an unbiased approach, we compared their initial behavior of player Bs and Cs in Experiment 1, Experiment 2, and the correlational study.

##### Coalition Size

As expected, we found significant differences in the frequency of BC coalition proposals across the three studies χ²(2) = 12.51, *p* = .001. The frequency did not differ between Experiment 2 (51.5%) and the correlational study (50.8%), *p* = .999, but it was significantly higher in Experiment 1 (61.2%) relative to both Experiment 2, *p* = .009, and the correlational study, *p* = .009. This suggests that the performance tips in Experiment 1 biased player Bs and Cs, see Figure 4.

##### Self-allocated Money

Similarly, the amount of money B/C allocated to themselves did not differ between Experiment 2 (*M* = 3,908 Euros, *SD* = 1,185) and the correlational study (*M* = 3,867 Euros, *SD* = 1,193), β = −40.81, 95% CI [−203.21, 121.60], *t*(860) = 0.49, *p* = .622. However, the allocation in Experiment 2 was significantly lower compared to Experiment 1 (*M* = 4,061 Euros, *SD* = 1,230), β = 193.60, [0.67, 304.92], *t*(860) = 2.34 *p* = .019, suggesting that the performance tips in Experiment 1 influenced how Bs and Cs allocated money to themselves. The regression model was statistically significant, F(2, 1339) = 3.24, p = .039, *R*^2^ > .01, see Figure 4.

Taken together, these analyses suggest that removing the performance tip did bring player Bs’ and Cs’ behavior back in line with how they behaved in a unmanipulated setting (Cantiani, Van Beest, Cruijssen, et al., 2024).

### Coalition Behavior

#### Partner-related Initial Behavior of Player A: Coalition Size

As shown in Figure 3, player As in the perspective-taking condition proposed more grand coalitions (48.3%) than those in the egocentric condition (35.8%). However, this difference was only marginally significant, *OR* = 1.68, 95% CI [1.00, 2.82], *z* = 1.96, *p* = .051). The deviance test comparing the null model to the model including the condition predictor was statistically significant, *X*^2^(1, 239) = 326.67, *p* = .049, *R*^2^ = .01.

#### Allocation-related Initial Behavior of Player A: Self-allocated Money

Again, player As in the perspective-taking condition allocated less money to themselves (*M* = 4,756 Euros, *SD* = 1,118) than those in the egocentric condition (*M* = 5,126 Euros, *SD* = 1,137), β = −369.58, 95% CI [−656.31, −82.86], *t*(238) = −2.54, *p* = .012, see Figure 3. The regression model was statistically significant, *F*(1, 238) = 6.45, *p* = .012, *R*^2^ = .03.

Table 3 shows that our manipulation of induced perspective-taking again led participants to propose more grand coalitions and to make less self-serving offers, replicating Experiment 1 and work on measured perspective-taking (Cantiani, Van Beest, Cruijssen, et al., 2024).

**Table 3.** Initial Behavior: Frequency of Opening Proposals and Associated Offers.

	Perspective-taking condition								
Position	Coalition	*N*	%	*M*_A_	*(SD)*	*M*_B_	*(SD)*	*M*_C_	*(SD)*
A (4 tons)	AB (7 tons)	16	13.3	5,218.8	(848.7)	3,781.2	(848.7)	—	—
	AC (6 tons)	46	38.3	5,756.6	(704.2)	—	—	3,243.4	(704.2)
	ABC (9 tons)	58	48.4	3,835.3	(543.8)	2,955.3	(177.8)	2,209.4	(514.3)
B (3 tons)	AB (7 tons)	14	11.7	4,857.2	(1,116)8)	4,142.8	(1116)	—	—
	BC (5 tons)	70	58.3	—	—	5,362.2	(590.2)	3,637.8	(590.2)
	ABC (9 tons)	36	30.0	3,488.9	(541.3)	3,066.7	(338.0)	2,444.4	(489.6)
C (2 tons)	AC (7 tons)	19	15.8	5,526.4	(920.0)	—	—	3,473.6	(920.0)
	BC (6 tons)	59	49.2	—	—	4,971.2	(505.8)	4,028.8	(505.8)
	ABC (9 tons)	42	35.0	3,683.3	(645.4)	2,997.6	(111.5)	2,319.1	(624.4)
	Egocentric condition								
	Coalition	*N*	%	*M*_A_	(*SD*)	*M*_B_	(*SD*)	*M*_C_	(*SD*)
A (4 tons)	AB (7 tons)	19	15.9	5,278.9	(775.0)	3,721.1	(775.0)	—	—
	AC (6 tons)	58	48.3	5,920.7	(798.8)	—	—	3,079.3	(798.8)
	ABC (9 tons)	43	35.8	3,986.1	(572.2)	2,911.6	(244.2)	2,102.3	(459.0)
B (3 tons)	AB (7 tons)	21	17.5	5,113.9	(1,026)	3,886.1	(1026)	—	—
	BC (5 tons)	58	48.3	—	—	5,282.8	(620.4)	3,717.2	(620.4)
	ABC (9 tons)	41	34.2	3,594.2	(487.7)	3,028.0	(157.3)	2,377.8	(484.7)
C (2 tons)	AC (7 tons)	21	17.5	5,285.73714.2	(916.1)	—	—	3,714.3	(916.1)
	BC (6 tons)	60	50.0	—	—	5,035.8	(378.8)	3,964.2	(378.8)
	ABC (9 tons)	39	32.5	3,387.2	(654.2)	2,974.3	(197.0)	2,638.5	(786.6)

### Coalition Outcomes

#### Partner-related Outcomes: Inclusion in Winning Coalitions

**Figure 5.**
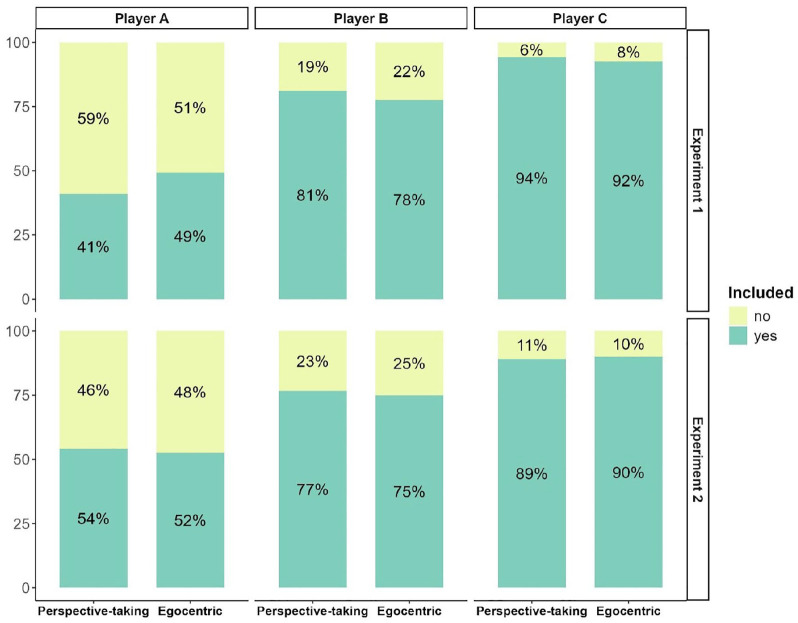
Inclusion rates in winning coalitions in Experiments 1 and 2.

Contrary to our predictions, the inclusion rates of player A did not differ significantly between the perspective-taking (54.2%) and egocentric (52.5%) conditions, *OR* = 1.07, 95% CI [0.64, 1.78], *z* = 0.26, *p* = .796 (see Figure 5). The deviance test comparing the null model to the model including the condition predictor was not significant, *X*^2^(1, 241) = 1.64, *p* = .795, *R*^2^ = .01.

Figure 5 also depicts the difference in inclusion rates between A, B, and C, reflecting the SiW effect, where players with more resources are typically excluded from coalitions. This shows that the Transport Game behaves as predicted by major coalition models and previous research (e.g., Wissink et al., 2021, 2022b).

#### Allocation-related Outcomes: Final Payoffs

**Figure 6.**
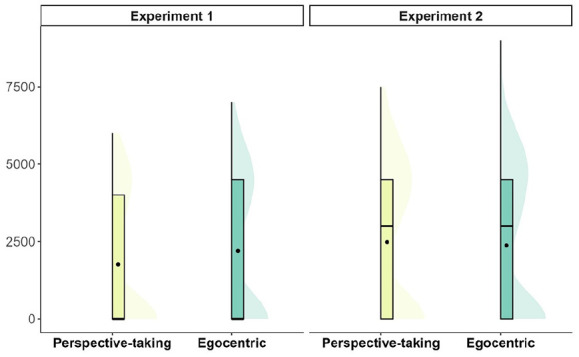
Final payoffs for Player A in Experiments 1 and 2. *Note.* Error bars indicate the 95% confidence intervals. Lines within the box plots indicate the median. Dots within the box plots indicate the mean.

Figure 6 shows that, contrary to our hypothesis, there were no significant differences in final payoffs between player As in the perspective-taking (*M* = 2,481 Euros, *SD* = 2,432) and egocentric condition (*M* = 2,377 Euros, *SD* = 2,446), β = 104.17, 95% CI [−516.10, 724.44], *t*(238) = 0.33, *p* = .741. The multiple regression model did not explain a significant proportion of variance, *F*(1, 238) = 0.11, *p* = .741, *R*^2^ = .00.

Table 4 summarizes these results and shows again that although both kinds of perspective-taking lead to similar coalition behaviors, at the outcome level, induced perspective-taking did not lead to the same benefits as measured perspective-taking in previous work.

**Table 4.** Coalition Outcomes: Frequency of Winning Coalitions and Associated Payoff.

Perspective-taking condition								
Coalition	*N*	%	*M*_A_	(*SD*)	*M*_B_	(*SD*)	*M*_C_	(*SD*)
AB (7 tons)	13	10.8	4,984.6	(533.6)	4,015.4 015.3	(533.6)	—	—
AC (6 tons)	28	23.3	5,335.7	(915.8)	—	—	3,664.3	(915.8)
BC (5 tons)	55	45.8	—	—	5,020.9	(420.5)	3,979.1	(420.5)
ABC (9 tons)	24	20.1	3,479.2	(499.5)	3,000	(0)	2,520.8	(499.5)
Egocentric condition								
Coalition	*N*	%	*M*_A_	(*SD*)	*M*_B_	(*SD*)	*M*_C_	(*SD*)
AB (7 tons)	12	10.0	5,191.7	(1576)	3,808.3	(1,576)	—	—
AC (6 tons)	30	25.0	5,163.3	(718.8)	—	—	3,836.6	(718.8)
BC (5 tons)	57	47.5	—	—	5,119.3	(368.6)	3,880.7	(368.6)
ABC (9 tons)	21	17.5	3,238.0	(436.4)	3,000	(0)	2,761.9	(436.4)

### Additional Variables

Experiment 2 aimed to address other potential explanations for the unexpected results in Experiment 1, focusing on reactive egoism (assessing perceived competitiveness about the setting and the interaction, fairness judgments and motivations) and individual differences in perspective-taking performance. Neither of these accounts was reflected in our data, which is why the findings are only briefly summarized here. The full analyses of these variables are reported in Supplemental Material B.

Regarding reactive egoism, we found that the game was perceived as more cooperative, rather than competitive, in the perspective-taking condition. Perspective-takers also showed higher motivation to minimize harm to others and lower motivation to maximize their own outcomes, contrary to reactive egoism predictions.

As for individual differences in perspective-taking performance, players in the perspective-taking condition showed greater accuracy in their estimations, although it is likely that the assessment of these estimations at the end of the experiment affected this measure.

### Exploratory Analyses

To address potential sample heterogeneity across the two experimental studies and the prior correlational study (Cantiani, van Beest, Cruijssen, et al., 2024), we conducted propensity score weighting to assess and correct for baseline differences (Olmos & Govindasamy, 2015). Using age, gender, and IRI scores for measured general perspective-taking and empathic concern as covariates, we found that the correlational sample was significantly older. After applying the Average Treatment Effect weighting procedure, covariate balance was achieved across all samples, and the weighted analyses replicated the confirmatory analyses (see Supplemental Materials B).

**Figure 7.**
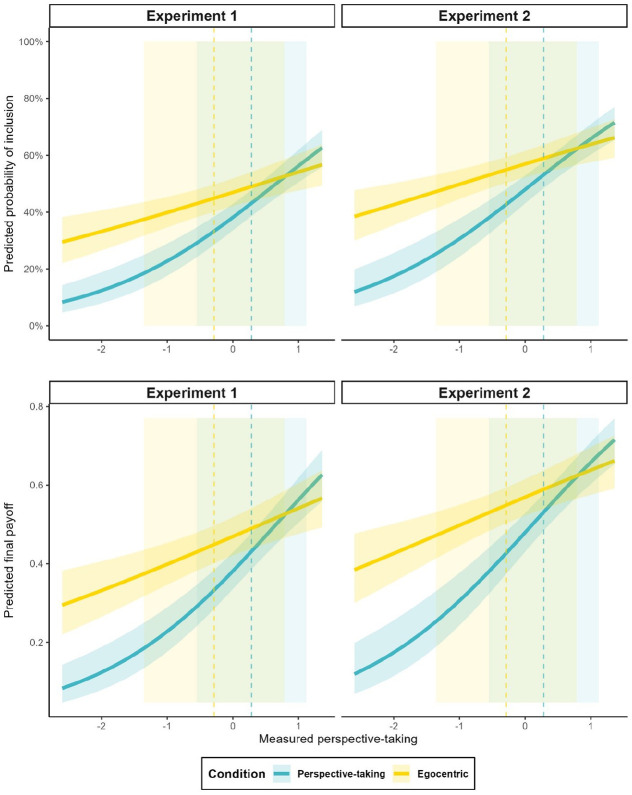
Predicted probability of coalition outcomes as a function of measured perspective-taking, by experimental condition. *Note*. Analyses use propensity score-weighted regression models (see Methods for weighting details; this figure depicts the first weighting approach). Shaded regions represent ±1 SD around condition-specific means (dashed vertical lines) of measured perspective-taking. Error bands show 95% confidence intervals around predictions.

We then focused on the experimental data alone (Experiments 1 and 2) to examine whether the effect of condition on negotiation outcomes depended on the extent to which participants self-reported engaging in perspective-taking during the game (i.e., measured perspective-taking). Using the balanced samples, we found a significant interaction between condition and measured perspective-taking for both inclusion in coalitions, *OR* = 1.58, 95% CI [1.24, 2.02], *p* < .001, and final payoffs, β = 554.4, [104.07, 1,004.82], *p* = .016. These results were consistent across different weighting strategies (see Supplemental Material B). While higher measured perspective-taking was associated with better outcomes across both conditions, this effect was stronger in the perspective-taking condition. However, the perspective-taking condition was less effective, and even detrimental, for individuals low in measured perspective-taking. At −1 SD, participants in the egocentric condition were significantly more likely to be included than those in the perspective-taking condition *OR* = 2.29, *p* < .001, and earned higher payoffs (estimated marginal mean difference: β = 951, *p* = .004). This advantage remained at average measured perspective-taking levels for inclusion, *OR* = 1.45, *p* = .001, although the difference in payoffs was not significant (β = 397, *p* = .073). At +1 SD, these differences disappeared: There was no difference in inclusion (*OR* = 0.92, *p* = .603) or in final payoffs (β = −157, *p* = .614) between conditions (see Figure 7).

## Discussion

The changes in our experimental design restored player B/C behavior to what was observed in the correlational study. Participants in the perspective-taking condition also again demonstrated good recall of the instructions they received and reported higher levels of perspective-taking during the negotiation. Furthermore, we replicated the effects of the perspective-taking induction on initial negotiation behavior, with player As in this condition proposing more grand coalitions and making more other-serving offers. Consistent with commonly reported prosocial effects of perspective-taking (e.g., Dovidio & Banfield, 2015; Galinsky et al., 2005), these players perceived the negotiation as less competitive, were more motivated to minimize harm, and focused less on maximizing their own payoffs. Although Experiment 2 solved the issues with player Bs and Cs in Experiment 1, this did not explain the lack of increased inclusion in winning coalitions or higher payoffs compared to previous research, nor did reactive egoism or perspective-taking performance.

However, once we accounted for preexisting differences across samples using propensity score weighting, a more nuanced pattern emerged: Induced perspective-taking led to better outcomes, but only for participants who reported high levels of measured perspective-taking. For those low in measured perspective-taking, receiving a perspective-taking tip was actually detrimental, potentially because the tip conflicted with their typical approach to negotiations. By contrast, participants who engaged in high levels of measured perspective-taking obtained better outcomes regardless of the tip, suggesting that it is the actual engagement of perspective-taking, not the manipulation alone, that drives successful outcomes. We will address this issue in more detail in the general discussion.

## General Discussion

Building upon prior work showing that measured perspective-taking predicts successful outcomes in coalition formation, we aimed to experimentally test its causal role using the same ecologically valid paradigm simulating horizontal transport collaboration in the transport sector. Across two high-powered experiments, we experimentally induced perspective-taking and found that it influenced participants’ initial behavior in line with the previous findings: Those induced to take others’ perspectives were more likely to propose grand coalitions and make other-serving offers. However, despite these behavioral shifts, induced perspective-taking did not lead to better outcomes. Participants in the perspective-taking condition were not included more often in winning coalitions, nor did they earn higher payoffs than those in the egocentric condition. To better understand this discrepancy, we systematically examined several potential explanations.

First, Experiment 2 addressed the issue with the neutral instructions given to Players B and C in Experiment 1, restoring their initial behavior to baseline levels. This, however, did not affect final coalition outcomes. Second, we did not find evidence for the idea that perspective-taking in competitive contexts can trigger more selfish behavior (reactive egoism; Epley et al., 2006). On the contrary, perspective-takers viewed the situation as less competitive, made more other-serving offers, and were more motivated to minimize harm to others rather than maximize personal gain. Thus, if anything, the manipulation reduced egoistic tendencies, which is also consistent with previous work (Cantiani, Van Beest, Cruijssen, et al., 2024). Lastly, we considered whether variation in perspective-taking performance might moderate the effectiveness of the manipulation. However, we found no evidence that participants who were more accurate in estimating other players’ behavior achieved better outcomes or benefited more from the manipulation. This null result should be interpreted cautiously, as our assessment of perspective-taking performance may have been influenced by the outcomes of the negotiation itself. Future work should continue exploring this possibility using more robust, task-independent measures.

However, related to the issue of perspective-taking performance, correcting for baseline differences between samples via propensity score weighting, we found that the effects of induced perspective-taking on coalition outcomes depended on measured perspective-taking levels: Participants who reported higher engagement perspective-taking achieved better outcomes, regardless of the condition they were assigned to, replicating previous findings (Cantiani, Van Beest, Cruijssen, et al., 2024). In contrast, participants who reported low perspective-taking engagement during the negotiation achieved worse outcomes when in the perspective-taking condition than in the egocentric condition. This suggests that induced perspective-taking may disrupt negotiation performance when it conflicts with participants’ disposition or willingness to adopt others’ perspectives. We will discuss the issue of inducing or measuring perspective-taking in more detail below.

### Theoretical and Practical Implications

Our findings have implications for coalition formation research. Major coalition formation theories assume that outcomes are determined solely by game parameters, with players perfectly anticipating others’ behaviors. This implicitly assumes flawless perspective-taking abilities across all individuals. However, building on prior research that showed how individual differences in perspective-taking predict coalition formation outcomes and behavior (Cantiani, Van Beest, Cruijssen, et al., 2024), we demonstrated that variation in perspective-taking can also be induced situationally, causally shaping coalition behavior, although not outcomes in this study. This once more demonstrates the importance of accounting for psychological variables, such as situational contexts or personality traits, to fully understand coalition negotiations. This introduces a new dimension to consider alongside traditional economic parameters (e.g., the 4-3-2 resource distribution of the current game). Future research should also explore further such variables and how they might interact with varying levels of perspective-taking. For instance, while high perspective-takers might exhibit more empathy and/or greater inferential capacities during coalition negotiations, low perspective-takers might rely more on power or dominance.

The present findings also contribute to perspective-taking research, particularly in the context of multiparty negotiations. Compared to prior work showing that in dyadic settings measured and induced perspective-taking lead to comparable effects (e.g., Galinsky et al., 2008; Galinsky, Wang, et al., 2008; Ku et al., 2010; Wang, Ku, et al., 2014; Wang, Kenneth, et al., 2014), these two ways of studying perspective-taking led to different results in coalition formation. As in a previous correlational study that measured perspective-taking (Cantiani, Van Beest, Cruijssen, et al., 2024), we found that inducing perspective-taking causes participants to approach more potential partners and shapes money allocation in a zero-sum negotiation context. However, only measured but not induced perspective-taking predicted coalition success. These findings suggest that perspective-taking operates differently in dyadic and multiparty negotiation settings.

This difference might reflect a conceptual distinction between measured and induced perspective-taking in the context of coalition formation. This view aligns with multidimensional models of perspective-taking, such as Gehlbach’s (2004) framework, which distinguishes between the *propensity* (volitional resources) to take others’ perspectives and the *ability* (cognitive resources) to do so. Measured perspective-taking may reflect both propensity and ability elements, whereas induced perspective-taking likely activates temporary motivation without matching existing dispositions. To illustrate, consider a marathon analogy: Measured perspective-taking resembles a person who regularly runs and enjoys doing so, while induced perspective-taking is like giving someone last-minute running tips before a race. The advice might be useful, but for someone unaccustomed to running, it may not improve performance or might even hinder it momentarily.

The interaction between induced and measured perspective-taking observed in the propensity weighting analysis supports this view. The perspective-taking instruction was not universally helpful; in fact, it appeared counterproductive for participants low in measured perspective-taking. These individuals may have experienced a mismatch between the instruction to engage in perspective-taking and their typical behavioral tendencies or motivational readiness to enact it. Individuals reporting high levels of measured perspective-taking, on the other hand, were successful irrespective of the condition they were assigned to, replicating previous works without situational inductions (Cantiani, Van Beest, Cruijssen, et al., 2024). Thus, the induction alone might not lead to success unless participants are already inclined or able to take others’ perspectives effectively.

The interpretation of the interaction between induced and measured perspective-taking also depends on how the measured perspective-taking scores are understood. If we treated measured perspective-taking as a pure state, unrelated to any trait dispositions, we could conclude that actual engagement in perspective-taking, regardless of the condition, is what improves outcomes. However, the fact that participants in the egocentric condition reported varying levels of measured perspective-taking and that low measured perspective-takers performed worse in the perspective-taking condition suggests the involvement of a trait component on top of the perspective-taking induction. This latter view is consistent with Whole Trait Theory (Fleeson & Jayawickreme, 2015). According to this account, each person has a distribution of perspective-taking states that varies as a function of their trait level and situational features (e.g., experimental instructions). Individuals higher on the trait are more likely to activate perspective-taking states, even without external prompts. Those low in measured perspective-taking may have encountered a mismatch with their natural approach when instructed to take others’ perspectives. This mismatch may have disrupted their behavior, reducing their effectiveness in the negotiation. In contrast, participants low in measured perspective-taking who were told to focus on themselves, consistent with their disposition, had better outcomes. This interpretation suggests that the effects of the perspective-taking manipulation depend on how well they align with an individual’s tendencies and willingness to enact it. When there is a mismatch, the instruction may not only fail to help but may also actively hinder coalition outcomes.

Lastly, our findings raised some questions about when and how reactive egoism may occur. Epley et al. (2006) showed that perspective-taking reduces egoism in judgment but increases it in behavior in a competitive context, but not in a cooperative one. However, our study, set in a similarly competitive context, did not replicate these effects. It appears that our paradigm may emphasize superordinate (shared) goals, making perspective-taking beneficial without incurring costs such as reactive egoism. Given these discrepancies, future research could further determine under what conditions perspective-taking is beneficial or detrimental in the context of coalition formation and other negotiations.

The current results have potential practical implications, particularly concerning the issue of low horizontal transport collaboration. Despite the differences in coalition outcomes, both induced and measured perspective-taking consistently lead to behaviors that appear conducive to CO_2_ reduction, that is, starting the negotiation by proposing larger coalitions. In the present study, these effects were observed in players with the most resources, who usually exhibit more self-serving judgments and behaviors (Wissink et al., 2021). This outcome may be attributed to perspective-taking altering participants’ perceptions of the setting, making it seem more cooperative and shifting motivations toward minimizing harm and away from maximizing personal profits. In a vacuum, these are exactly the perceptions and behaviors most conducive to combating the human contribution to climate change.

However, decisions about cargo space use are primarily driven by profitability. Since induced perspective-taking did not produce economic benefits, and may even be disadvantageous for some, its practical value is limited unless it aligns with business incentives. A promising approach relates to norm implementation. Research shows that specific norms can guide coalition behavior, and when such norms are in place, perspective-taking no longer offers an additional advantage (Cantiani, Van Beest, & Erle, 2024). This opens the door for designing norms that align sustainable behavior with profitability, making them more appealing to businesses. Achieving this alignment will require collaboration between psychologists and policymakers to ensure that perspective-taking behaviors also lead to economic gain.

### Limitations and Future Research

Several factors may have weakened the effects of the perspective-taking manipulation and deserve attention in future research. One potential factor is the strength and timing of the manipulation itself. In both experiments, participants received the perspective-taking prompt only once before the start of the negotiations. While this had an immediate effect on their initial offers, the effect appeared to diminish in later rounds (see Supplemental Material B for details). Future research could test whether reinforcing the prompt at the beginning of each round might prolong its impact and ultimately lead to stronger effects on final outcomes. On balance, it is likely that participants’ behavior during each round of coalition formation plays a more decisive role in shaping outcomes than a one-time prompt at the outset. This stresses the need to study real-time interactions and actual behavior in social psychological research on negotiations.

A second consideration is the online nature of the experimental setting, which may have limited participants’ engagement with the perspective-taking instructions. However, the incentivized design likely encouraged attention, and across all studies, participants showed high levels of comprehension, attentiveness, and behavior consistent with the manipulations. Given these patterns, this explanation appears less likely, though it cannot be fully ruled out.

A third potential factor is the structural disadvantage faced by player A due to the SiW effect. Previous research has shown that this disadvantage can be reduced by applying interventions across all group members, for instance, by promoting norms of deservingness (Wissink et al., 2023). In contrast, our manipulation targeted only player As. While this may have influenced their individual behavior, it may not have been sufficient to shift overall group dynamics. Prior studies of perspective-taking in small group contexts often assigned them to different treatments (e.g., Epley et al. 2006; Gilin et al., 2013; Trötschel et al., 2011). Future research should explore whether prompting all triad members to engage in perspective-taking more effectively encourages the formation of larger, more inclusive coalitions. Furthermore, future studies should include a control condition to provide a baseline, which may clarify whether the effects observed are driven specifically by perspective-taking, egocentrism, or a combination of both.

Another possible factor relates to participants’ experience of exclusion. Prior research has shown that feeling ostracized motivates efforts to reconnect with others, often by focusing more on social rather than self-related information (Maner et al., 2007; Hess & Pickett, 2010). This attending to others’ perspectives may help restore social connection (Knowles, 2014). Because player A is most often excluded in our setup, it is possible that participants in the egocentric condition also spontaneously engaged in perspective-taking, which could have reduced the difference between conditions. However, participants in both conditions reported feeling similarly ostracized (see Supplemental Material B), making this explanation unlikely based on the present data.

Finally, further research is needed to better understand how perspective-taking operates in group contexts. It remains unclear whether participants consider each group member’s perspective separately or treat the group as a whole. The increased tendency of perspective-takers to propose grand coalitions may not only reflect a strategy to avoid exclusion but could also stem from considering multiple perspectives simultaneously. Future studies could explore this by instructing participants to take the perspective of one partner versus both. Taking a single perspective may encourage smaller coalitions focused on that partner, while considering multiple perspectives might lead to broader, more inclusive outcomes. These insights could inform strategies to promote inclusive decision-making in group negotiations, including sustainability efforts.

## Supplemental Material

sj-docx-1-psp-10.1177_01461672251349706 – Supplemental material for Measured but Not Induced Perspective-taking Predicts Success in Coalition Formation Supplemental material, sj-docx-1-psp-10.1177_01461672251349706 for Measured but Not Induced Perspective-taking Predicts Success in Coalition Formation by Anabela Cantiani, Ilja van Beest and Thorsten M. Erle in Personality and Social Psychology Bulletin

sj-docx-2-psp-10.1177_01461672251349706 – Supplemental material for Measured but Not Induced Perspective-taking Predicts Success in Coalition Formation Supplemental material, sj-docx-2-psp-10.1177_01461672251349706 for Measured but Not Induced Perspective-taking Predicts Success in Coalition Formation by Anabela Cantiani, Ilja van Beest and Thorsten M. Erle in Personality and Social Psychology Bulletin

## Notes

1. These include minimum resource theory (Gamson, 1961), bargaining theory (Komorita & Chertkoff, 1973), pivotal power theory (Gamson, 1961), weighted probability theory (Komorita, 1974). For extensive reviews of classic coalition formation theories, see Kahan & Rapoport (1984); Komorita, 1984; Murnighan, 1978.

2. While many theories in social decision-making more broadly—such as behavioral game theory (Camerer, 2003), models of bounded rationality (Simon, 1955), and social identity theory (Tajfel & Turner, 1979)—have challenged the rationality axiom and emphasized the role of psychological and contextual factors, these approaches have rarely been applied to coalition formation. One exception is the Social Utility Model by Van Beest and Van Dijk (2007), which proposes that players’ decisions in coalitions are influenced not only by self-interest but also by fairness concerns. However, this model is descriptive rather than predictive and does not fundamentally question the assumption that players possess full and accurate information about others’ mental states.

3. While these findings align with the social utility approach to coalition formation proposed by Van Beest and Van Dijk (2005), which challenged the rational self-interest assumption by classic coalition theories, showing that players also consider fairness, our focus differs. We are not primarily concerned with prosocial motivations, but rather with strategic adaptation in coalition behavior. Specifically, we argue that the strategies typically prescribed by major coalition formation theories—such as forming minimal winning coalitions and making self-serving offers—do not always lead to successful outcomes.
