# Supplementary material for: Measured but Not Induced Perspective-taking Predicts Success in Coalition Formation
Source: Pers Soc Psychol Bull. 2025 Jul 14;52(9):2824–42. doi: 10.1177/01461672251349706 (PMC13392153; doi:10.1177/01461672251349706)
Supplement: sj-docx-1-psp-10.1177_01461672251349706 – Supplemental material for Measured but Not Induced Perspective-taking Predicts Success in Coalition Formation [file sj-docx-1-psp-10.1177_01461672251349706.docx]

**Supplemental Material A: Detailed Description of All Study Manipulations and Measures**

This supplemental material describes all manipulated variables and measures used in the main study in full detail.

# Experiment 1

## Manipulated Variables

The manipulation was implemented after players had learned the game scenario and successfully answered comprehension questions, but immediately before the start of negotiations. Players received specific instructions framed as a 'performance tip' for the upcoming coalition negotiation, adapted from previous research by Gilin et al. (2013) and Trötschel et al. (2011).

In the *perspective-taking* condition, player As' instructions emphasized taking the perspective of other players: “*Previous research on negotiations has demonstrated that an effective strategy to maximize outcomes is to take the other participants’ perspectives and to avoid focusing too much on your own perspective. Try to focus on the other participants’ perspective, such as the other participants’ intentions and interests in the negotiation. Focusing on the other participants’ perspectives will help you successfully achieve your goals*.”. Unlike Trötschel et al. (2011), who also manipulated prosocial versus egoistic motivations by instructing participants to either "maximize joint outcomes" or "maximize individual outcomes" we deliberately omitted such wording to avoid influencing participants' motivations.

In the *egocentric* condition, player As' instructions emphasized focusing on one's own perspective: “*Previous research on negotiations has demonstrated that an effective strategy to maximize outcomes is to concentrate one's own perspective and to avoid getting distracted by the other participants' perspectives. Try to focus on your own perspective, such as your intentions and interests in the negotiation. Focusing on your own perspective will help you successfully achieve your goals*.” (Trötschel et al., 2011).

In both the perspective-taking and the egocentric conditions, player Bs and Cs received neutral instructions that were matched in length and complexity to the experimental manipulations, but neither emphasized perspective-taking or egocentrism: “*Previous research on negotiations has demonstrated that an effective strategy to maximize outcomes is to decide on your own approach and to follow it consistently and to avoid switching strategies too much. Try to focus on following your own approach consistently. Focusing and following your own approach will help you successfully achieve your goals*.” (Gilin et al., 2013).

Lastly, to reinforce the manipulation, following Gilin et al. (2013), we asked all players to write a short essay about their “performance tip”. For the perspective-taking condition: “*Please, take the perspective of the other participants and describe their intentions and interests in the negotiation*:” For the egocentric condition: “*Please, focus on your own perspective and describe your intentions and interests in the negotiation*:” For the neutral condition: “*Please, describe your approach*:”.

## Comprehension and Attention Checks

Players completed four multiple choice quizzes to verify whether they understood game instructions. When players gave a wrong answer, they were presented with the correct option and justification. They were then asked to answer the same question until they answered correctly. This was done to ensure that every player who entered the negotiation understood the rules. The questions were: 1) *What company do you represent*? (company A/B/C); 2) *How many tons does your company transport to Paris every day*? (My company transports 4/3/2 tons); 3) *What is the bonus paid if the big truck is used*? (8000/9000/10000 Euro); 4) *What coalitions could use the big truck (don't forget to also name the ones you are not in)*? (AB, AC/AB, BC/AC, BC/AB, AC, BC/AB, AC, BC, ABC).

To check players' attentiveness in completing the questionnaires at the end of the study, we introduced an item in which players were instructed to select "5" as a response.

## Measured Variables

After the completion of the game, we assessed several variables to evaluate the effectiveness of our manipulation and to explore key effects of perspective-taking as shown by previous research. These variables were presented to participants in the order described below.

### Manipulation Check I: Recall conditions

Our first manipulation check aimed to verify whether participants could correctly recall the instructions they received. Participants identified which 'performance tip' they received before the negotiation, choosing from three options: 1) “*Take the other participants' perspectives*”, 2) “*Concentrate on one's own perspective*”, or 3) “*Decide on your own approach*”. Player Bs and Cs were expected to select option 3, as they received neutral instructions. For player As, selections were considered correct when choosing option 1 in the perspective-taking condition and option 2 in the egocentric condition.

### Measured General Perspective-Taking

Players completed the 7-items perspective-taking subscale of the Interpersonal Reactivity Index (IRI; Davis, 1980), as an index of their general disposition to engage in perspective-taking. The items were: “*I find it difficult to see things from another person's point of view*” (reverse coded), “*I always try to look at a problem from all sides before making a decision*”, “*I try to understand my friends better by imagining how they view things*”, “*If I am sure about something, I do not waste a lot of time listening to other people's arguments*” (reverse coded), “*I take into account that others view things differently*”, “*If I am angry with someone, I usually try to look at the situation from his/her side for a while*”, “*Before criticizing somebody, I try to imagine how I would feel if I were in their place*”.

This is a Likert-type scale ranging from 1 (“Does not describe me well”) to 5 (“Describes me very well”).

### Empathic Concern

Players also completed the 7-items subscale “Empathic concern” of the IRI (Davis, 1980), as an index of their general tendency to experience sympathy and compassion for unlucky others. The items were: “*I often have tender, concerned feelings for people less fortunate than me*”, “*Sometimes I don't feel very sorry for other people when they are having problems*” (reverse coded), “*When I see someone being disadvantaged, I get a nasty feeling*”, “*I usually don't care much about other people's unhappiness*” (reverse coded), “*When I see someone being treated unfairly, I have little compassion*” (reverse coded), “*I am often touched by what other people go through*”, “*I am a gentle person*”.

This is a Likert-type scale ranging from 1 (“Does not describe me well”) to 5 (“Describes me very well”).

Both IRI scales served as control measures, as we hypothesized no differences in IRI perspective-taking or empathic concern between conditions.

### Measured Contextualized Perspective-Taking

Our second manipulation check aimed to verify the extent to which participants reported engaging in perspective-taking. We used the contextualized perspective-taking scale (Cantiani, Van Beest, Cruijssen et al., 2024), consisting of 4 items measuring participants' disposition to adopt other players' psychological viewpoints during the negotiation. This contextualized the assessment of perspective-taking in two ways: a) it captures players’ tendency to adopt their game partner’s point of view (i.e., particular targets), and b) it focuses on this specific setting (i.e., particular situational context). In line with prior research, the scale showed higher reliability (α = 0.93) than the IRI perspective taking scale (α = 0.78), indicating it is a psychometrically sound measure of contextualized perspective-taking.

We hypothesized that player A in the perspective-taking condition would score higher than player Bs and Cs, and higher than player A in the egocentric condition. Conversely, we expected player A in the egocentric condition to score lower than player Bs and Cs in the same condition.

The items were introduced with the statement "*Think back to your thoughts during the negotiation and answer the following questions*:" as participants filled out the scale after the negotiation to refer to the situation accurately. The items were: “*During the negotiation, I tried to take other players’ perspectives*”, “*During the negotiation, I imagined how the other players were feeling*”, “*During the negotiation, I made an effort to see the world through the other players’ eyes*”, “*During the negotiation, I sought to understand players’ viewpoints*”.

This is a Likert-type scale ranging from 1 (“Does not describe me well”) to 5 (“Describes me very well”).

### Inclusion of Other in the Self (IOS, Aron et al., 1992)

Participants indicated the extent to which they perceive similarity and connection between themselves and each of the two players by shifting the circles that represent themselves and their respective partner. The items were introduced with the statement “*In this task we ask you to shift the circles to choose a pair of circles that best represents your relationship with the other participants. The overlap of the circles represents the similarity and connection you feel towards the other participant. For example, a stronger connection and similarity between you and the other participant would be represented by a greater overlap of the circles. Which configuration best describes your relationship with the participant representing Company [A/B/C]?”*

### Satisfaction (Galinsky et al., 2008)

Participants indicated how satisfied they are with the way they were treated during the negotiation by the player they chose to form a coalition with on a 5-point Likert scale (1 = “Not satisfied at all”, 5 = “Very satisfied”). The statement was “*Please indicate how satisfied you are with the way you were treated during the negotiation by the player you chose to form a coalition with*”.

### Need Threat Scale (Williams, 2009)

Participants indicate how satisfied were across the four basic needs proposed by the need-threat model (i.e., belonging, control, self-esteem and meaningful existence) on a 5-point Likert scale (1 = “Do not agree”, 5 = “Totally agree”). The items were introduced with the statement “*Based on your experience during the negotiation, please indicate the extent to which you agree with each of the following statements:*”. The items were: “*I felt as one with the other players*”, “*I had the feeling that I belonged to the group during the negotiation*”, “*I did not feel accepted by the other players*”, “*During the negotiation, I felt connected with one or more other players*”, “*I felt like an outsider during the negotiation*”, “*I felt that I received offers from the other players as often as I wanted*”, “*I felt in control over the negotiation*”, “*I had the idea that I affected the course of the negotiation*”, “*I had the feeling that I could influence the direction of the negotiation*”, “*I had the feeling that the other players decided everything*”, “*The negotiation made me feel insecure*”, “*I had the feeling that I failed during the negotiation*”, “*I had the idea that I had the same value as the other players*”, “*I was concerned about what the other players thought about me during the negotiation*”, “*I had the feeling that the other players did not like me*”, “*During the negotiation, it felt as if my presence was not meaningful*”, “*I think it was useless that I participated in the negotiation,” “I had the feeling that my presence during the negotiation was important*”, “*I think that my participation in the negotiation was useful*”, and “*I believed that my contribution to the negotiation did not matter.*”

### Demographics

Lastly, we collected demographic information on gender identification and age.

# Experiment 2

## Procedure

The experimental design in Experiment 2 was almost identical to Experiment 1, with one key difference: only player A received a performance tip containing the manipulation of perspective-taking. Player Bs and Cs did not receive any performance tip, neutral or otherwise. This change was implemented to isolate the effect of the perspective-taking manipulation on player A and to prevent unintended influences on player Bs and Cs' behavior.

We made several adjustments to our final measurements after the experiment. The first manipulation check was modified to address only player As. Player As were asked to recall the performance tip they received, with only two response options corresponding to the perspective-taking or egocentric conditions. This change aligned with the modified experimental design where only player A received the manipulation. Although our preregistration included asking all participants whether they received a performance tip, we realized in hindsight that this recall might be problematic for player Bs and Cs, who did not receive any tip. Consequently, we decided to ask only player A about the tip they received.

To capture individual differences in performance, we added a perspective-taking accuracy measure where participants estimated initial proposals and coalition outcomes. Regarding the initial proposals, participants were asked to estimate how much money on average was allocated in initial proposals to each player in each coalition. They did this separately for each player position (e.g., “*How much money will player A allocate to themselves in an AB, AC, ABC coalition*”, “*How much money will player B allocate to themselves in AB, BC, ABC coalitions*”, and so on). In addition, we asked participants to estimate the ratio of coalitions formed at the end of the task (e.g., how many AB, AC, BC, ABC coalitions will be formed if this game is played repeatedly).

To track signs of reactive egoism, we included measures of: perceived competitiveness, fairness judgments (Epley et al., 2006) and motivations (Wissink et al., 2023). First, participants rated the perceived competitiveness of the game setting. Second, they rated how cooperative or competitive they perceived the game to be before the actual negotiation started. Next, we assessed participants’ fairness judgements. Lastly, we assessed player’s motivations to 1) “*Maximize your own outcomes*”, 2) “*Minimize harm to the other bargainers*”, and 3) “*Make sure that every bargainer got what they deserved*”.

## Measured Variables

### Perspective-Taking Accuracy

Estimation of Proposals. Participants were asked to estimate how much money on average was allocated in initial proposals to each player in each coalition. They did this separately for each player position (e.g., “*How much money will player A allocate to themselves in an AB, AC, ABC coalition*”, “*How much money will player B allocate to themselves in AB, BC, ABC coalitions*”, and so on). The exact instructions were as follows: “*We are conducting this experiment with hundreds of participants. Are you able to predict what people would allocate for themselves, on average, in their first proposed coalitions?*

*Please complete the table provided below, indicating how much you think participants in the role of Company A, Company B, and Company C would allocate to themselves in each of the possible coalitions they could propose.*

*For example, in the first row, we start with the AB coalition. Your goal is thus to predict how much player As, on average, would want if they were to propose such a coalition. Next, you fill in how much player Bs, on average, would want if they were to propose such an AB-coalition. The cell corresponding to player Cs is implied to be not applicable in the AB coalition context*.”

We operationalized perspective-taking accuracy by computing the absolute difference between the estimations made by participants and the actual proposals, with larger values indicating the less accuracy. For instance, if a participant estimated that player Bs would allocate to themselves, on average, 5100 in a BC-coalition, and the actual mean self-allocated proposal was of 5500, the absolute difference was of 400 euros.

Estimation of Outcomes. In addition, we asked participants to guess the ratio of coalitions formed at the end of the task (e.g., how many AB, AC, BC, ABC coalitions will be formed if this game is played repeatedly). The accuracy rates of outcome estimation were operationalized as with the proposal estimation, and higher values indicate less accuracy. The specific instructions were: “*Remember that we are conducting this experiment with hundreds of participants. Are you able to predict the frequency (%) of formed coalitions? Please indicate the frequency (%) of formation for each of the four possible coalitions in the table below. You may continue to the next page once the total adds up to 100%.*”

We operationalized perspective-taking (in)accuracy by computing the absolute difference between the estimations made by participants and the actual outcomes, with larger values indicating less accuracy. For instance, if a participant estimated that BC coalitions would form in 30% of final coalitions, but it actually was formed in 50% of final coalitions, the absolute difference was of 20. We added the absolute differences for between estimations and actual coalition frequencies for all coalition types to quantify how well participants anticipated what would happen during the negotiation. Lower scores on this variable indicated higher accuracy.

### Perceived Competitiveness (Epley et al., 2006)

### **About the Setting.** After finishing the negotiation, participants rated how competitive they perceived the game setting on a Likert-scale ranging from 1 (very cooperative) to 10 (very competitive). Specifically, we asked: “At the beginning of the study, we gave you the following instructions:

*The companies transport 9 tons every day from Amsterdam to Paris. Company A transports 4 tons, Company B transports 3 tons, and Company C transports 2 tons. A minimum of 5 tons is needed to use the big truck and thereby claim the bonus of 9000 euros. Companies are competitors with each other. Your company wants you to get the best possible deal for your business.*

*Please indicate how “cooperative” (working together) or “competitive” (competing against each other) you perceived this situation* ***BEFORE*** *the actual negotiation started:”*

About the Interaction. After finishing the negotiation, we asked “*Now, please indicate how “cooperative” (working together) or “competitive” (competing against each other) you felt the other participants were* ***DURING*** *the actual negotiation*:” They answered using a Likert-scale ranging from 1 (very cooperative) to 10 (very competitive).

### Fairness Judgements (Epley et al., 2006)

Participants were asked: "*For the following options, what would you consider to be a fair share for your company to get out of the total premium of 9000 euros?*" They provided responses for each possible coalition they could form depending on their own position. For instance, player As respond this question in relation to AC, AB and ABC, but not in relation to BC.

### Motivations (Wissink et al., 2023)

Participants were asked: “*Please think back to your motivations during bargaining and answer the next questions. There are no right or wrong answers. During bargaining, to what extent were you motivated to: 1) Maximize your own outcomes, 2) Minimize harm to the other bargainers, and 3) Make sure that every bargainer got what they deserved*”. They rated each statement on a 7-point Likert scale (1 = “Not at all”, 7 = “Very much”).
