# Supplementary material for: Measured but Not Induced Perspective-taking Predicts Success in Coalition Formation
Source: Pers Soc Psychol Bull. 2025 Jul 14;52(9):2824–42. doi: 10.1177/01461672251349706 (PMC13392153; doi:10.1177/01461672251349706)
Supplement: sj-docx-2-psp-10.1177_01461672251349706 – Supplemental material for Measured but Not Induced Perspective-taking Predicts Success in Coalition Formation [file sj-docx-2-psp-10.1177_01461672251349706.docx]

# Supplemental Materials B: Additional Analyses

This supplementary material presents additional analyses that we have carried out and that have not been included in the main text.

# Experiment 1

## Manipulation and Comprehension Checks

Below we present the results from comprehension checks and manipulation checks.

***Comprehension Checks***

Most participants demonstrated a strong understanding of the game rules: 94.1% answered all comprehension questions correctly, 5.1% made one mistake, and only 0.8% made two mistakes.

***Manipulation Check I: Recall Condition***

Player As accurately recalled their assigned condition at high rates: 94.4% (115/122) in the perspective-taking condition and 83.3% (100/120) in the egocentric condition. For player Bs and Cs, 74.2% (359/484) correctly recalled receiving neutral instructions. The recall was notably higher for player As in the perspective-taking condition. This may reflect the greater distinctiveness and cognitive engagement elicited by these instructions compared to the more default-aligned egocentric and neutral conditions.

***Manipulation Check II: Measured Individual Differences***

We performed separate two-way analysis of variance (ANOVA) with player position (A, B, C) and condition as between-subjects factors on measured contextualized perspective-taking, measured general perspective-taking, and empathic concerns. The latter dispositional scales served as control measures, which we expected to remain stable and unaffected by the experimental manipulation, consistent with Whole Trait Theory (Fleeson et al., 2021), which holds that global traits represent stable averages over time. In contrast, measured contextualized perspective-taking (Cantiani, Van Beest, Cruijssen, et al., 2024), was designed to capture momentary, situational activations of perspective-taking specifically within the coalition formation task. This scale reflects the state-like expression of trait perspective-taking as it fluctuates with task demands. Accordingly, we predicted player As in the perspective-taking/egocentric conditions to score higher/lower than player Bs and Cs in their respective conditions. In addition, we predicted player As in the perspective-taking condition to score higher than player As in the egocentric condition.

**Measured Contextualized Perspective-taking.** The measured contextualized perspective-taking demonstrated high internal consistency (α = 0.93). The main effect of player position was not statistically significant, *F*(2, 719) = 0.40, *p* = .672, but the main effect of condition was significant, *F*(1, 719) = 7.03, *p* = .008. As anticipated, an interaction between condition and player position qualified this main effect, *F*(2, 719) = 7.27, *p* < .001. Holm’s post-hoc comparisons showed that scores for player Bs and Cs did not vary significantly between conditions (*p* = .422 and *p* = .484, respectively), whereas player A scores did (*p* < .001). Player As in the perspective-taking condition engaged in perspective-taking to a greater extent (*M* = 3.93, *SD* = 0.85) than player As in the egocentric condition (*M* = 3.38, *SD* = 0.94). Furthermore, within the perspective-taking condition, player A reported higher scores than player C (*M* = 3.58, *SD* = 1.05; *p* = .002). There were no significant differences between player As and B (*M* = 3.75, *SD* = 0.84; *p* = .099), nor between B and C (*p* = .136). Within the egocentric condition, no differences were found after p value corrections between player A and B (*M* = 3.65, *SD* = 1.00; *p* = .089), or C (*M* = 3.67, *SD* = 0.99; *p* = .089), nor between B and C (*p* = .913).

As expected, the scores on the other two more general dispositional scales, namely measured general perspective-taking and empathic concern, did not differ between conditions.

#### Measured General Perspective-taking. The internal consistency was adequate, with a Cronbach's alpha of α = 0.78. The analysis revealed that neither the main effect of player position, *F*(2,719) = 0.16, *p* = .854, nor the main effect of condition, *F*(1,719) = 1.41, *p* = .236, were statistically significant. Additionally, the interaction between player position and condition was not statistically significant, *F*(2,719) = 2.78, *p* = .063.Top of FormBottom of Form

#### Empathic Concern. The scale showed high internal consistency, with a Cronbach's alpha of α = 0.86. The results indicated that the main effect of player position was statistically significant, *F*(2,719) = 5.85, *p* = .003. In contrast, the main effect of condition was not significant, *F*(1, 719) = 0.02, *p* = .886, and the interaction between player position and condition also did not reach significance, *F*(2,719) = 0.89, *p* = .413.

Post-hoc analyses using pairwise t-tests with Holm's correction for multiple comparisons revealed that player As (*M* = 3.87, *SD* = 0.73, *p* = .001) and Bs (*M* = 3.78, *SD* = 0.75, *p* = .033) reported significantly higher scores of empathic concern than player Cs (*M* = 3.64, *SD* = 0.74). There were no significant differences between player As and Bs (*p* = .213).

## Participant Exclusion

Excluding participants who did not correctly answer all comprehension questions (*n* = 9), the attention check (*n* = 3), or manipulation check I (*n* = 27) did not affect the results reported in the main manuscript. The data and analysis scripts used to run these checks are openly available in the project’s OSF repository for full transparency and reproducibility.

## Coalition Behavior

### Grand Coalition Proposal and Selection Across Rounds

We also explored whether the preference for grand coalitions persisted during the negotiation and after player As realized other players' preferences. We found that the proportion of grand coalitions in the remaining rounds of negotiation (i.e., excluding the first round and averaging the rest) both proposed and selected by player As in the perspective-taking condition (proposal: *M* = 0.51, *SD* = 0.50; selection: *M* = 0.54, *SD* = 0.50) did not differ significantly from those the egocentric condition (proposal: *M* = 0.51, *SD* = 0.50, β = 0.00, *95%CI* [-0.14, 0.13], *p* = .952; selection: *M* = 0.47, *SD* = 0.50; β = 0.08, *95%CI* [-0.06, 0.21], *p* = .257).

Furthermore, the initial proposals made by player As seemed to have influenced player Bs and Cs. We observed that player Bs and Cs proposed and selected significantly more grand coalitions (averaging all negotiation rounds, excluding the first) in the perspective-taking condition (proposal: *M* = 0.51, *SD* = 0.50; selection: *M* = 0.54, *SD* = 0.50) compared to the egocentric condition (proposal: *M* = 0.38, *SD* = 0.49, β = 0.14, *95%CI* [0.04, 0.23], *p* = .005; selection: *M* = 0.40, *SD* = 0.49, β = 0.14, *95%CI* [0.05, 0.24], *p* = .003).

## Negotiation Variables

### Negotiation Duration

We explored whether player As in the perspective-taking condition needed more time to reach an agreement than player As in the egocentric condition. In line with previous research, players in the perspective-taking condition needed more rounds, on average, to reach an agreement (*M* = 2.13, *SD* = 2.22) than players in the egocentric condition (*M* = 1.73, *SD* = 1.02; β = 0.40, *95%CI* [0.15, 0.65], *p* = .002). The linear regression model, explained a significant proportion of variance, *F*(1, 724) = 9.61, *p* = .002, *R^2^* = .01.

### Partner Size (SiW)

We explored whether player As in the perspective-taking condition were more likely than player As in the egocentric condition to approach weak players (player C) than strong players (player B). The DV ‘strength is weakness’ was dummy coded as 1 = ‘approach weak players’ and 0 = ‘approaching strong players’ in the first proposals, excluding proposed grand coalitions.

A logistic regression analysis showed no significant effect of condition on the likelihood of approaching weak players, *OR* = 0.90, *95%CI* [0.39, 2.08], *p* = .802.

### Flexibility

We explored whether player As in the perspective-taking condition were more likely than player As in the egocentric condition to exhibit flexible behavior (i.e., they might be less likely to stick to their own economic proposals, and/or to the same partner).

The DV ‘flexibility in proposal’ was dummy coded as 1 = ‘selecting own proposal’ and 0 = ‘selecting a different proposal’. A logistic regression analysis showed no significant effect of condition on the likelihood of selecting the same partner that was originally approached, *OR* = 1.13, *95%CI* [0.60, 2.14], *p* = .699.

The DV ‘flexibility in partner selection ’ was dummy coded as 1 = ‘selecting same player’ and 0 = ‘selecting another player’. A logistic regression analysis showed no significant effect of condition on the likelihood of selecting the same partner that was originally approached, *OR* = 1.02, *95%CI* [0.47, 2.20], *p* = .961.

### Concessions

We explored whether player As in the perspective-taking condition were more likely than player As in the egocentric condition to make concessions (i.e., to accept other’s economic offers even if these are less advantageous for themselves). The DV ‘concessions’ was computed by subtracting self-allocated money from a proposed offer (phase I, first round) to a selected offer (phase II, first round).

A linear regression analysis indicated that there was no significant difference in concessions between the perspective-taking and egocentric conditions, β = -138.71, *95%CI* [-316.40, 38.98], *p* = .125.

## Psychological Variables

### Need Threat Scale (Williams, 2009)

We explored whether (1) those who are not included in a winning coalition are worse off in terms of need satisfaction than those who are included. We also explored if this was moderated by (2a) player position (i.e., perhaps strong player As are more hurt by exclusion than weak players, and by (2b) condition (i.e., whether those in the perspective-taking condition are more affected by exclusion).

A multiple linear regression was conducted to examine these relationships. The model included inclusion, player position, and condition as predictors, as well as interaction terms between inclusion and player position, and between inclusion and condition. The model explained 47% of the variance in need satisfaction, *R*² = .47, *F*(7, 714) = 90.47, *p* < .001. Results indicated a significant main effect of inclusion, β = 1.27, SE = 0.10, *p* < .001, suggesting that participants included in a winning coalition reported higher need satisfaction (*M* = 3.77, *SD* = 0.60) compared to those who were not included (*M* = 2.44, *SD* = 0.75). There were no significant main effects of player position (Player B: β = 0.14, SE = 0.11, *p* = .181; Player C: β = -0.11, SE = 0.17, *p* = .504) or condition, β = -0.02, SE = 0.09, *p* = .795. Furthermore, no significant interaction effects were found between inclusion and player position (Player B: β = 0.06, SE = 0.13, *p* = .676; Player C: β = 0.19, SE = 0.19, *p* = .298) or between inclusion and condition, β = -0.03, SE = 0.11, *p* = .816.

### Inclusion of Other in the Self (IOS, Aron et al., 1992)

We examined whether player As in the perspective-taking condition reported greater connection and similarity toward player Bs and Cs than player As in the egocentric condition. The results of a Welch Two-Sample t-test revealed no significant differences between the IOS scores reported by A in the perspective-taking (*M* = 2.26, *SD* = 1.61) and the egocentric (*M* = 2.05, *SD* = 1.30) conditions (*t*(461.6) = -1.55, *95%CI* [-0.47, 0.06] , *p* = .123).

We also explored whether player Bs and Cs in the perspective-taking condition reported greater connection and similarity towards A than player Bs and Cs in the egocentric condition. The results of a Welch Two-Sample t-test revealed no significant differences between the perspective-taking (*M* = 1.86, *SD* = 1.27) and the egocentric (*M* = 1.92, *SD* = 1.34) conditions (*t*(479.56) = 0.51, *95%CI* [-0.17, 0.29], *p* = .612).

### Perceived Satisfaction (Galinsky et al., 2008)

We explored whether player Bs and Cs in the perspective-taking condition reported greater satisfaction during the negotiation than player Bs and Cs in the egocentric condition. The results of a Welch Two-Sample t-test revealed no significant differences in satisfaction scores between the perspective-taking (*M* = 4.11, *SD* = 0.99) and the egocentric (*M* = 4.09, *SD* = 1.00) conditions (*t*(481.1) = -0.14, *95%CI* [-0.23, 0.20], *p* = .891).

# Experiment 2

## Manipulation and Comprehension Checks

Below we present the results from comprehension checks and manipulation checks.

***Comprehension Checks***

Again, most participants understood the game well. 92.4% answered all comprehension questions correctly, 6.1% made one mistake, and only 1.53% made two or three mistakes.

***Manipulation Check I: Recall Condition***

Player As accurately recalled their performance tip at high rates: 94.2% (113/120) in the perspective-taking condition and 86.7% (104/120) in the egocentric condition. As in Experiment 1, recall was higher in the perspective-taking condition, likely due to the greater distinctiveness and cognitive effort associated with this instruction compared to the more default-aligned egocentric tip.

***Manipulation Check II: Measured Individual Differences***

As in Experiment 1, we performed separate two-way analysis of variance (ANOVA) with player position (A, B, C) and condition as between-subjects factors on measured contextualized perspective-taking, measured general perspective-taking, and empathic concerns. See further details in the corresponding section of Experiment 1.

**Measured Contextualized Perspective-taking.** The contextualized perspective-taking scale demonstrated high internal consistency, with a Cronbach's alpha of α = 0.93. The analysis showed that both the main effects of player position, *F*(2, 712) = 5.57, *p* = .004, and condition *F*(1, 712) = 6.70, *p* = .010, were statistically significant. As expected, they were qualified by an interaction between condition and player position, *F*(2, 712) = 10.67, *p* < .001.

Holm’s post-hoc comparisons revealed that scores for player Bs and Cs did not vary significantly between conditions (*p* = .443 and *p* = .992, respectively), whereas player A scores did (*p* < .001). As expected, player As in the perspective-taking condition engaged in perspective-taking to a greater extent (*M* = 3.91, *SD* = 0.86) than player As in the egocentric condition (*M* = 3.31, *SD* = 1.08). However, within the perspective-taking condition, there were no significant differences between player A and B (*M* = 3.77, *SD* = 0.94; *p* = .223), player A and C (*M* = 3.87, *SD* = 0.85; *p* = .709), nor between B and C (*p* = .399). Within the egocentric condition, player A showed significantly lower scores than B (*M* = 3.86, *SD* = 0.81; *p* < .001) and C (*M* = 3.87, *SD* = 0.77; *p* < .001), while no differences were found between B and C (*p* = .943).

As in Experiment 1, the scores for both the perspective-taking and empathic concern scales on the IRI did not differ between conditions (see details below).

### Measured General Perspective-taking. The scale demonstrated a high internal consistency, with a Cronbach's alpha of α = 0.81. The analysis revealed that neither the main effect of player position, F(2,712) = 0.88, p = .416, or condition, F (1,712) = 0.16, p = .694, no significant interaction between these factors, F (2,712) = 0.15, p = .858.

### Empathic Concern. The scale demonstrated a high internal consistency, with a Cronbach's alpha of α = 0.86. The analysis revealed that neither the main effect of player position, F (2,712) = 0.24, p = .787, or condition, F (1,712) = 0.16, p = .685, no significant interaction between these factors, F (2,712) = 1.86, p = .157.

## Participant Exclusion

Excluding participants who did not correctly answer all comprehension questions (*n* = 14), the attention check (*n* = 2), or the manipulation check I (*n* = 23) did not alter the reported results in the main manuscript. The data and analysis scripts used to run these checks are openly available in the project’s OSF repository for full transparency and reproducibility.

## Coalition Behavior

### Grand Coalition Proposal and Selection Across Rounds

The analysis revealed no significant differences in the proportion of grand coalitions proposed or selected by player As in the perspective-taking condition compared to the egocentric condition (proposal: β = 0.04, *95%CI* [-0.08, 0.19], *p* = .410; selection: β = 0.03, *95%CI* [-0.10, 0.16], *p* = .619). Additionally, no significant differences were observed in the proposals and selections of player Bs and Cs. Although player A’s initial proposals seemed to influence player Bs and Cs, the proportion of grand coalitions proposed and selected by player Bs and Cs in the perspective-taking condition did not differ significantly from those in the egocentric condition (proposal: β = -0.01, *95%CI* [-0.11, 0.08], *p* = .775; selection: β = -0.04, *95%CI* [-0.13, 0.06], *p* = .437).

## Negotiation Variables

### Negotiation Duration

Contrary to previous findings, we did not find significant differences between the number of rounds needed to reach an agreement in the perspective-taking condition (*M* = 1.88, *SD* = 1.54) relative to the egocentric condition (*M* = 2.02, *SD* = 1.90, β = -0.15, *95%CI* [-0.40, 0.10], *p* = .246). The linear regression model did not explain a significant proportion of variance, *F*(1, 718) = 1.35, *p* = .245, *R*^2^ = .00.

### Partner Size (SiW)

A logistic regression analysis showed no significant effect of condition on the likelihood of approaching weak players, *OR* = 0.94, *95%CI* [0.44, 2.05], *p* = .879.

### Flexibility

A logistic regression analysis showed no significant effect of condition on the likelihood of selecting the same partner that was originally approached, *OR* = 0.74, *95%CI* [0.37, 1.46], *p* = .392, nor on the likelihood of selecting the same partner that was originally approached, *OR* = 0.81, *95%CI* [0.45, 1.43], *p* = .464.

### Concessions

The DV ‘concessions’ was computed as in Experiment 1. A linear regression analysis indicated that there was no significant difference in concessions between the perspective-taking and egocentric conditions in the first round, β = -164.17, *95%CI* [-379.60, 51.26], *p* = .135.

We also explored concessions at the end of the negotiation. The DV ‘concessions’ was computed by subtracting self-allocated money from the last proposed offer (phase I, last round) to the last selected offer (phase II, last round). A linear regression analysis revealed a main effect of condition on concessions, β = -246.67, *95%CI* [-449.14, -44.19], *p* = .017, so that player As in the perspective-taking condition made larger concessions in the final round than player As in the egocentric conditions.

**Additional Variables**

***Perceived Competitiveness***

We explored whether player As’s perceptions of competitiveness about the negotiation setting, and the interaction varied between conditions.

**About the Setting.** We found that the game setting was perceived as less competitive in the perspective-taking condition (*M* = 6.10, *SD* = 2.36) than in the egocentric condition (*M* = 6.35, *SD* = 2.41), β = -0.81, *95%CI* [-1.42, -0.20], *p* = .009.

**About the Interaction.** We found no significant differences in perceptions about the interaction between the perspective-taking condition (*M* = 6.02, *SD* = 2.46) and the egocentric condition (*M* = 5.96, *SD* = 2.49), β = 0.07, *95%CI* [-0.54, 0.69], *p* = .814.

***Fairness Judgments***

We examined whether player A's perceptions of fair allocations differ (a) between conditions, and (b) from the actual allocations they assigned to themselves.

To test (a), we first averaged the three values provided for each coalition option (AB, AC, ABC). No significant difference in fairness judgments was found between conditions, β = -84.39, *95%CI* [-265.57, 96.79], *p* = .361. We then analyzed only the judgments corresponding to coalitions player As actually proposed initially. For example, if a participant proposed an ABC coalition, only their fairness judgment for the ABC coalition was analyzed. This revealed a non- significant effect of condition, β = -317.65, *95%CI* [-644.57, 9.27], *p* = .057, with perspective-taking player As deeming it fair to receive less (*M* = 4588.51, *SD* = 1195.94) compared to player As in the egocentric condition (*M* = 4906.16, *SD* = 1353.16).

To test (b), we computed the difference between fairness judgments and actual self-allocations for proposed coalitions. For instance, if a participant proposed an ABC coalition allocating 3800 to themselves, and indicated 4000 as fair for an ABC coalition, the difference would be -200 Euros. This analysis showed no significant differences between conditions, β = 40.31, *95%CI* [-180.70, 261.33], *p* = .720.

***Motivations***

We explored whether the following motivations differed for player As across conditions.

**Minimize Harm.** Player As in the perspective-taking condition expressed higher motivations to minimize harm to other bargainers (*M* = 4.07, *SD* = 2.76) than those in the egocentric condition (*M* = 3.56, *SD* = 1.95), β = 0.50, *95%CI* [0.03, 0.98], *p* = .037.

**Every Player Gets What They Deserve.** We found no significant differences between the perspective-taking condition (*M* = 5.02, *SD* = 1.75) and the egocentric condition (*M* = 4.73, *SD* = 1.82), β = 0.29, *95%CI* [-0.17, 0.74], *p* = .220.

**Maximize Own Outcomes.** Player As in the perspective-taking condition expressed lower motivations to maximize own outcomes (*M* = 5.29, *SD* = 1.44) than those in the egocentric condition (*M* = 5.80, *SD* = 1.25), β = -0.51, *95%CI* [-0.85, -0.17], *p* = .004.

***Perspective-Taking Performance***

Estimation of Proposals. We investigated whether player As in the perspective-taking condition made fewer errors in estimating average behavior at the beginning of the negotiation compared to those in the egocentric condition. Additionally, we explored whether this relationship was moderated by the extent to which players reported engaging in perspective-taking (i.e., measured perspective-taking). To test this, we conducted a linear regression with condition (perspective-taking vs. egocentric), measured perspective-taking, and their interaction as predictors of proposal estimation accuracy. Neither the main effect of induced perspective-taking, β = -54.19, *95%CI* [-1618.49, 1510.11], *p* = . 946, nor measured perspective-taking, β = 60.36, *95%CI* [-832.13, 952.86], *p* = .894, nor their interaction, β = -170.38, *95%CI* [-1610.91, 1270.15], *p* = .816, were significant in predicting accuracy.

**Estimation of Outcomes.** Using the same model, we assessed accuracy in estimating outcomes. The interaction term between condition and measured perspective-taking was not significant, β = -1.47, *95%CI* [-8.89, 5.95], *p* = .696. However, we found that the estimations of final coalitions presented fewer errors in the perspective-taking than in the egocentric condition, β = -8.90, *95%CI* [-16.96, -0.85], *p* = .030. Surprisingly, measured perspective-taking negatively predicted accuracy, β = 7.57, *95%CI* [2.98, 12.17], *p* = .001. This alerted us to the possibility that one’s negotiation outcome might influence these estimations, as they were assessed at the end of the study. We repeated the analysis, adding inclusion in winning coalitions (yes vs. no) and its interaction with the other variables to the model. Indeed, inclusion was the only significant predictor of accuracy, β = 26.77, *95%CI* [15.08, 38.46], *p* < .001. None of the other predictors reached statistical significance: condition, β = −0.86, *95%CI* [−12.92,11.20], *p* = .889, measured perspective-taking, β = 1.71, *95%CI* [−4.91,8.34], *p* = . 610, the interaction between condition and measured perspective-taking, β = 2.44, *95%CI* [−7.66,12.53], *p* = .635, the interaction between condition and inclusion, β = −10.48, *95%CI* [−26.59,5.63] , *p* = .201, the interaction between measured perspective-taking and inclusion, β = 6.33, *95%CI* [−2.70,15.37], *p* = .169, and the three-way interaction, β = −7.87, *95%CI* [−22.71,6.96], *p* = .297.

**Propensity Score Weighting**

To account for potential samples biases, propensity score weighting was performed using the covariates age, gender, IRI perspective-taking scores^[[1]](#footnote-1)^, and IRI empathic concern scores. Pre-weighting diagnostics revealed differences in baseline characteristics across samples. Particularly, age varied significantly between samples, with the correlational sample (of the previous study) being older on average compared to the experimental groups (Experiment 1 and 2). Additionally, there were differences in empathic concern and perspective-taking tendencies, while gender distribution was relatively balanced.

To correct for pre-existing differences between samples, we used the WeightIt package in R to compute weights based on the Average Treatment Effect (ATE) method. Nine participants with missing covariate data were excluded from this process. After weighting, diagnostic checks confirmed that covariate balance was successfully achieved across all variables (Olmos & Govindasamy, 2015). Specifically, all standardized mean differences (SMDs) including age, which was initially imbalanced, fell below the expected threshold of 0.1. This indicates that the weighting procedure solved the imbalance between samples (see Table S1).

We further assessed covariate balance visually using the Cobalt package in R (Figure S1). In addition, a weighted multinomial logistic regression predicting study membership from covariates showed no significant associations (all *ps* > .11), confirming that the groups were statistically comparable after weighting. However, it is important to note that the effective sample size for the correlational study group decreased substantially after weighting (from 567 to ~364), due to higher variability in weights. This reduction reflects greater variability in the assigned weights, suggesting that fewer individuals in the correlational sample closely matched participants in the experimental conditions in terms of the covariates.

| **Table S1** |  |  |  |
| --- | --- | --- | --- |
| *Balance Diagnostics and Effective Sample Sizes After Propensity Score Weighting* | | | |
| Covariates | Max. Std. Difference |  |  |
| Age | 0.059 |  |  |
| Gender | 0.069 |  |  |
| IRI PT Score | 0.033 |  |  |
| IRI EC Score | 0.036 |  |  |
| Sample |  | Unadjusted ESS | Adjusted ESS |
| Experiment 1 |  | 726 | 580.63 |
| Experiment 2 |  | 720 | 699.91 |
| Correlational |  | 567 | 363.81 |
| *Note*. ESS = Effective Sample Size; IRI PT = Measured General Perspective-Taking; IRI EC = Empathic Concern. Maximum standardized mean differences (Max. Std. Difference) below 0.10 indicate good balance. All adjusted ESS values reflect the precision loss due to weighting.  **Figure S1**  *Covariate Balance Before and After Weighting*  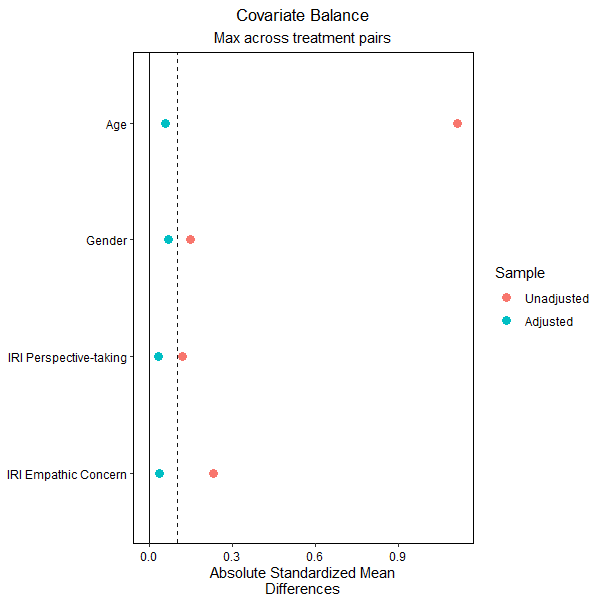 | | | |

We re-ran the main analyses examining inclusion in winning coalitions and final payoffs using weighted (logistic) regression. We modeled the correlational study separately from the experimental studies (Experiments 1 and 2) to include the respective predictors (standardized measured perspective-taking, IRI empathic concern, and IRI perspective-taking scores) for the correlational study and only condition for the experimental studies). The data of the experimental studies was combined in these analyses. As shown in Table S2, the weighted model results aligned with the original findings.

**Table S2**

*Results of Outcome Analyses Using Propensity Score-Weighted Models*

| **Correlational Study** | | | | |
| --- | --- | --- | --- | --- |
|  | **Inclusion** | | | |
| *Predictors* | *Odds Ratios* | | *CI* | *p* |
| (Intercept) | 2.72 | | 2.46 – 3.01 | **<0.001** |
| Measured PT mean | 1.52 | | 1.37 – 1.67 | **<0.001** |
| IRI EC mean | 0.75 | | 0.67 – 0.83 | **<0.001** |
| IRI PT mean | 1.06 | | 0.94 – 1.18 | 0.327 |
| Observations | 567 | | | |
| R^2^ Tjur | 0.036 | | | |
|  | **Final Payoffs** | | | |
| *Predictors* | *Estimates* | | *CI* | *p* |
| (Intercept) | 3015.63 | | 2846.26 – 3184.99 | **<0.001** |
| Measured PT mean | 292.96 | | 124.75 – 461.17 | **0.001** |
| IRI EC mean | -263.14 | | -433.60 – -92.69 | **0.003** |
| IRI PT mean | 82.87 | | -107.46 – 273.21 | 0.393 |
| Observations | 567 | | | |
| R^2^ / R^2^ adjusted | 0.033 / 0.028 | | | |
| **Experimental Studies (1 and 2)** | | | |  |
|  | **Inclusion** | | |  |
| *Predictors* | *Odds Ratios* | *CI* | *p* |  |
| (Intercept) | 1.00 | 0.86 – 1.17 | 0.968 |  |
| condition [Perspective-taking] | 0.88 | 0.71 – 1.09 | 0.238 |  |
| Observations | 482 | | |  |
| R^2^ Tjur | 0.001 | | |  |
|  | **Final Payoffs** | | |  |
| *Predictors* | *Estimates* | *CI* | *p* |  |
| (Intercept) | 2275.65 | 1968.44 –  2582.86 | **<0.001** |  |
| condition [Perspective-taking] | -186.01 | -611.95 –  239.92 | 0.391 |  |
| Observations | 482 | | |  |
| R^2^ Tjur | 0.001 | | |  |

Furthermore, we investigated whether participants' inclusion in winning coalitions and their final payoffs were influenced not only by the experimental manipulation but also by their self-reported engagement in perspective-taking during negotiations (measured perspective-taking), using balanced samples. We conducted weighted logistic and linear regression analyses focusing on Player A data from the two experimental studies (combined) and employed two different propensity score weighting strategies to ensure robust estimates.

In the first approach, we used the original weights derived from comparisons across all three samples, including the correlational sample. These weights adjusted for differences in demographic and trait variables (age, gender, IRI PT, and EC scores) between the experimental and non-experimental samples. This model^[[2]](#footnote-2)^ revealed a significant interaction between condition and measured perspective-taking for both inclusion in coalitions, *OR* = 1.58, 95% CI [1.24, 2.02], *p* < .001, and final payoffs, β = 554.4, 95% CI [104.07, 1004.82], *p* = .016. (see Figure S2). To inspect the interaction further, we examined the simple slopes of measured perspective-taking within each condition. In the egocentric condition, measured perspective-taking was positively associated with inclusion, *OR* = 1.32, 95% CI [1.14, 1.52], *p* < .001. This association was stronger in the perspective-taking condition, *OR* = 2.08, 95% CI [1.71, 2.54], *p* < .001. A different pattern was observed for final payoffs. In the egocentric condition, measured perspective-taking was not significantly associated with final payoffs, β = 173.9, 95% CI [–111.4, 459.2], *p* = .232. In contrast, in the perspective-taking condition, measured perspective-taking positively predicted higher final payoffs, β = 728.3, 95% CI [381.9, 1074.7], *p* < .001. Overall, higher measured perspective-taking was associated with better outcomes across both conditions, and this effect was more pronounced in the perspective-taking condition, and consistently regarding inclusion outcomes.

However, comparisons between conditions at low, average, and high measured perspective-taking showed that the perspective-taking condition was less effective, and even detrimental, for individuals low in measured perspective-taking. At –1 SD, participants in the egocentric condition were significantly more likely to be included than those in the perspective-taking condition *OR* = 2.29, *p* < .001, and earned higher payoffs (estimated marginal mean difference: β = 951, *p* = .004). This advantage remained at average measured perspective-taking levels for inclusion, *OR* = 1.45, *p* = .001, although the difference in payoffs was not significant (β = 397, *p* = .073). At +1 SD, these differences disappeared: there was no difference in inclusion (*OR* = 0.92, *p* = .603) or in final payoffs (β = –157, *p* = .614) between conditions. This suggests the perspective-taking manipulation only leads to favorable outcomes when participants actually engage in perspective-taking to a high extent, and it can be detrimental for those who are not able or willing to engage in perspective-taking.

**Figure S2**

*Predicted Probability of Coalition Outcomes as a Function of Measured Perspective-taking by Experimental Condition*

**
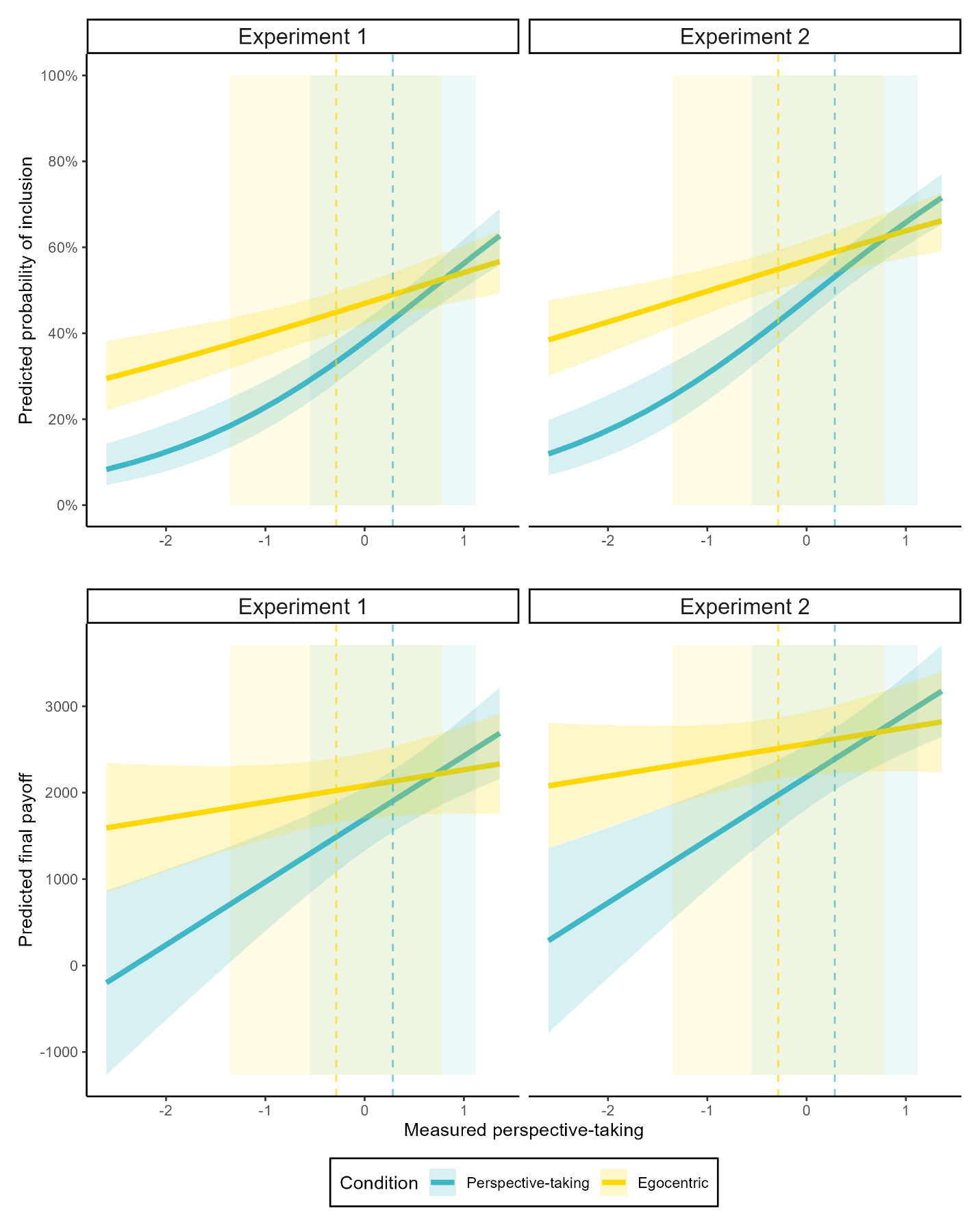
**

*Note*. Analyses use propensity score-weighted regression models (this figure depicts the first weighting approach). Shaded regions represent ±1 SD around condition-specific means (dashed vertical lines) of measured perspective-taking. Error bands show 95% confidence intervals around predictions.

To more precisely isolate experimental effects, we conducted a second weighting strategy focused solely on participants from the experimental samples (Experiments 1 and 2). Although participants were randomly assigned to conditions, we checked whether covariates differed by condition. Pre-weighting diagnostics confirmed no significant differences between conditions, consistent with randomization. However, we found that covariate distributions differed significantly between the two experiments, namely by sample. To address this, we re-estimated propensity scores using the same covariates (i.e., age, gender, and IRI PT and EC scores) this time to balance across Experiment 1 and Experiment 2. Post-weighting diagnostics indicated successful balance, with all covariate associations with study assignment showing *p* > .90 (see Table S3 and Figure S3). We then re-ran the outcome models using these within-experiment weights^[[3]](#footnote-3)^. Once again, as depicted in Figure S4, we found a significant interaction between condition and measured perspective-taking scores for inclusion (*OR* = 1.40, 95%CI [1.08, 1.83], *z* = 2.48, *p* = .013), and for final payoffs (β = 433.8, 95%CI [19.84, 847.73], *t*(476) = 2.06, *p* = .040).

Together, these analyses indicate that the effect of the condition on negotiation outcomes is contingent on participants’ actual engagement in perspective-taking (measured perspective-taking) during the task, and that this effect is consistent across different weighting strategies and levels of sample adjustment.

| **Table S3** |  |  |  |
| --- | --- | --- | --- |
| *Balance Diagnostics and Effective Sample Sizes After Propensity Score Weighting* | | | |
| Covariates | Max. Std. Difference |  |  |
| Age | -0.002 |  |  |
| Gender | 0.003 |  |  |
| IRI PT Score | -0.001 |  |  |
| IRI EC Score | -0.001 |  |  |
| Sample |  | Unadjusted ESS | Adjusted ESS |
| Experiment 1 |  | 726 | 697.82 |
| Experiment 2 |  | 720 | 699.97 |
| *Note*. ESS = Effective Sample Size; PT = Perspective-Taking; EC = Empathic Concern. Maximum standardized mean differences (Max. Std. Difference) below 0.10 indicate good balance. All adjusted ESS values reflect the precision loss due to weighting. | | | |

**Figure S3**

*Covariate Balance Before and After Weighting*


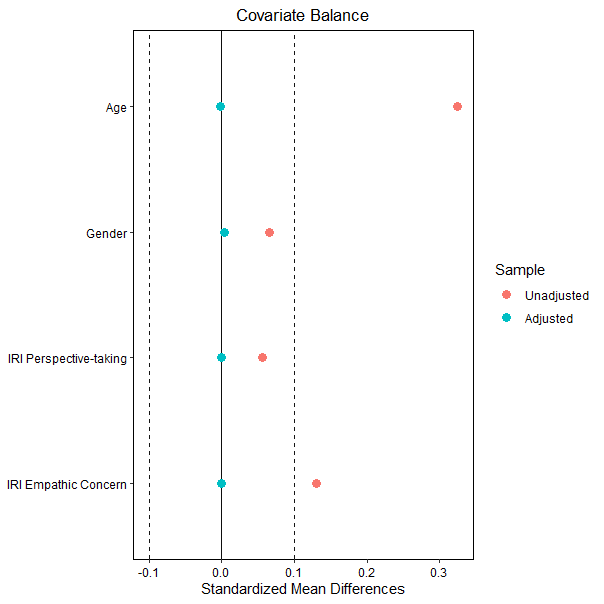


**Figure S4**

*Predicted Probability of Coalition Outcomes as a Function of Measured Perspective-taking by Experimental Condition*

**
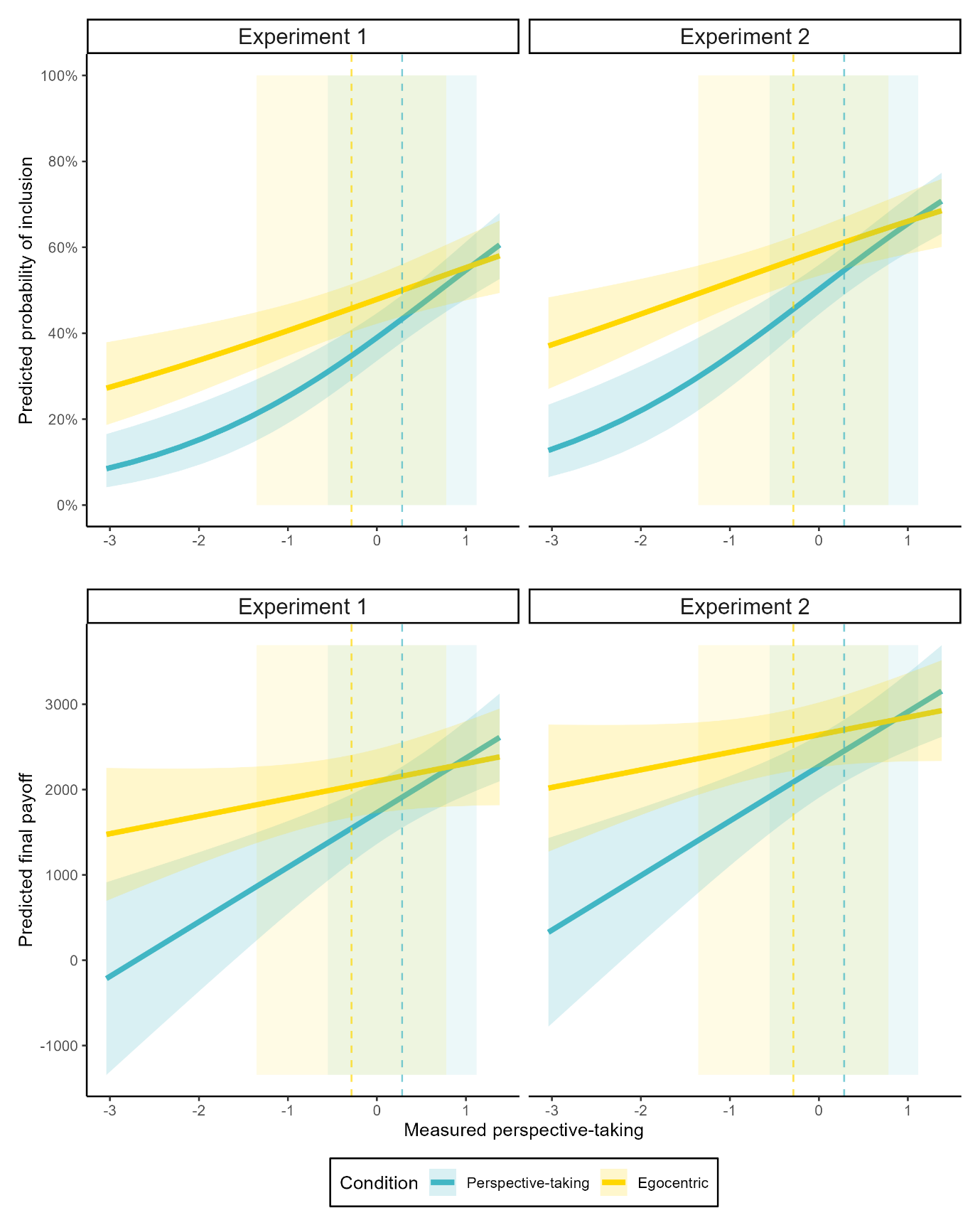
**

*Note*. Analyses use propensity score-weighted regression models (this figure depicts the second weighting approach). Shaded regions represent ±1 SD around condition-specific means (dashed vertical lines) of measured perspective-taking. Error bands show 95% confidence intervals around predictions**.**

1. Note on terminology: To reduce confusion, we clarify terminology used throughout the supplement. The IRI perspective-taking scale is referred to as measured general perspective-taking, while the contextualized perspective-taking measure used during the negotiation task is referred to as measured perspective-taking. The latter reflects trait expression in context and is the primary predictor of interest in these analyses. [↑](#footnote-ref-1)
2. The analyses reported here were conducted on the combined dataset from Experiments 1 and 2 to maximize statistical power and reliability. However, the figure displays results separately for each experiment to illustrate that the observed trends were consistent across both samples. [↑](#footnote-ref-2)
3. The analyses reported here were conducted on the combined dataset from Experiments 1 and 2 to maximize statistical power and reliability. However, the figure displays results separately for each experiment to illustrate that the observed trends were consistent across both samples. [↑](#footnote-ref-3)
